# Stabilizing features of cefotaximase harbouring plasmids enable persistence in UK livestock

**DOI:** 10.1099/mgen.0.001775

**Published:** 2026-07-07

**Authors:** Nicholas Duggett, Daisy E. Gates, Manal AbuOun, Jeremy Chanter, Chris Teale, Javier Nunez-Garcia, Muna F. Anjum

**Affiliations:** 1 Animal and Plant Health Agency, Addlestone, UK; 2 Animal and Plant Health Agency, Thirsk Veterinary Investigation Centre, Thirsk, UK; 3 Animal and Plant Health Agency, Starcross Veterinary Investigation Centre, Exeter, UK; 4 Animal and Plant Health Agency, Veterinary Investigation Centre, Shrewsbury, UK

**Keywords:** antimicrobial resistance, extended spectrum beta lactamases, livestock, mobile genetic elements, plasmids

## Abstract

Extended-spectrum cephalosporinases (ESCs) confer resistance to a range of beta-lactam compounds, including many cephalosporins and have grown in prevalence since the early 2000s. ESCs are often disseminated via plasmids, which can also encode other genes that confer bacterial fitness. Until recently, detailed characterization of plasmids encoding antimicrobial resistance genes was limited by the technical ability to close plasmid genomes. Recent advances in long-read technology have simplified this problem, facilitating the characterization of the genetic structure of plasmids and their component genes. This study examined the genetic mechanisms that underpin the stability of the most common cefotaximase-encoding plasmids in *Escherichia coli* recovered from UK livestock between 2013 and 2020. The most common plasmid replicon types were IncF, IncI1 and IncX, and the predominant cefotaximases were *bla*_CTX-M-1_, *bla_CTX-M-14_*, *bla*_CTX-M-15_ and *bla*_CTX-M-55_; in this study, we focused on IncI1 plasmids only which were most commonly associated with the cefotaximases in our dataset. Comparison of circularized plasmid genomes revealed that IncI1/*bla*_CTX-M-1_ plasmids showed a high degree of genetic similarity to one another in the time period examined, as did IncI1/*bla*_CTX-M-14_ plasmids, indicating stability of these plasmid genomes. Overall, our results indicate that IncI1/*bla*_CTX-M-1_ plasmids carry genes promoting their maintenance in host *E. coli* and encode several genes that may enhance their fitness. Therefore, these plasmids may play an important role in the persistence of ESC resistance in *E. coli* isolated from One-Health compartments, despite nationwide reductions in cephalosporin usage in both humans and livestock.

**Impact Statement**

Antimicrobial resistance (AMR) poses a mounting global health threat, with extended spectrum beta-lactamase producing *Escherichia coli* increasingly implicated in resistance to critically important antimicrobials. This study leverages long-read sequencing to characterise cefotaximase-harbouring plasmids in *E. coli* from UK livestock and meat between 2013–2020, revealing how plasmid architecture and stability mechanisms contribute to the persistence and dissemination of resistance genes. By linking genomic data to national AMR surveillance, the findings offer vital insights into the evolutionary dynamics of epidemic plasmids and inform risk assessment strategies in the context of reduced antimicrobial usage. This work strengthens the role of genomic epidemiology in guiding stewardship and safeguarding public health.

## Data Summary

Assembled chromosomal and plasmid sequence accessions are JBREGC000000000-JBREGH000000000, PV686651-PV686689 and PX236808-PX236810 (also listed in Table 1). All short-read sequencing data generated by previous studies and used in this study are available under accession numbers PRJEB34493, PRJEB67810 and PRJEB89252 (further details in Table S3).

## Introduction

Bacterial resistance is a burgeoning issue, and we are rapidly exhausting our arsenal of available antimicrobials [1,2]. Antimicrobial resistance (AMR) infections are currently estimated to be responsible for 1.27 million deaths per annum globally; a figure expected to rise to ~10 million by 2050 if no action is taken [3,4]. The WHO categorises certain classes of antimicrobials as the highest priority critically important antimicrobials (HP-CIAs) for human health. At present, these are: third and fourth generation cephalosporins, including extended spectrum cephalosporins, fluoroquinolones, fosfomycin and colistin [5]. In the UK, this includes harmonized monitoring of AMR in healthy livestock and retail meat as continuation of the European Directive 2003/99/EC [6,7], which the UK has continued nationally since leaving the EU.

Extended-spectrum cephalosporinases (ESCs) mediate cephalosporin resistance through hydrolysis of the beta-lactam ring and have grown in prevalence since the early 2000s [8,10]. In recent years, the frequency of SHV and TEM-based extended spectrum beta lactamases (ESBL) has declined, and a new group of class A ESBLs, the CTX-M ESBLs (cefotaximases), has been increasing in prevalence [11]. Unlike the plasmid origin of TEM and SHV, CTX derived from chromosomal beta-lactamases of *Kluyvera spp* [12], close relatives of *Escherichia coli. bla*_CTX-M-1,_*bla*_CTX-M-14_ and *bla*_CTX-M-15_ are among the most widely distributed cefotaximases and often disseminated amongst STs in humans and animals, often found integrated chromosomally or on plasmids [11,13].

The antimicrobial treatment of livestock is a known driver of AMR in Enterobacterales [14,15]. For example, in 2014, 23.4% of *E. coli* originating from UK pig farms were ESC producers [16]. Tighter control on antimicrobial usage has been in place over the last decade in recognition that usage increases AMR and as part of antibiotic stewardship. In 2015, a collaborative report by the European Food Standards Agency, European Center for Disease Control and European Medicines Agency demonstrated positive associations between antimicrobial consumption and resistance in both humans and animals [17]. In the UK, the usage of cephalosporins in pigs has been reduced by 90% since 2014, from 0.19 mg kg^−1^ to around 0.02 mg kg^−1^ [18] and their use in poultry is prohibited by the British Poultry Council. There has been a reduction in circulating resistances as seen by the significant decrease in the prevalence of ESBL-producing *E. coli* from retail chicken samples from 65.4% to just 12% between 2014–2022 [19]. Analyses from the same study revealed that meat samples without skin were more likely to exhibit an ESC phenotype, indicating that contamination might occur during the processing of meat. Genomic AMR characterization of isolates from this surveillance identified groups of closely related isolates sharing the same AMR genotype between different surveillance years and sources [20].

Plasmids play a role in the dissemination of AMR, including HP-CIAs, between unrelated *E. coli* in food, livestock and humans. In fact, there is a wealth of studies that demonstrate the contribution of plasmids to the maintenance and transmission of ESCs. For example, a study examining ESC producing *E. coli* from pigs, collected as part of harmonized AMR monitoring in the UK, showed that an ST131 *bla*_CTX-M-27_ subclade was present in two healthy pig caeca, which clustered in a clade with human isolates and had characteristics that mirrored those of highly pathogenic ST131 isolates that have been prevalent in extra-intestinal human infections [21]. This and other studies highlight that detailed characterization of ESBL encoding plasmids is warranted due to their importance in transmission of AMR genes between and within compartments [22,25].

As autonomous genetic elements, plasmids are in a constant co-evolutionary battle with their host and, as such, exhibit adaptations to ameliorate the cost of their carriage and promote stability [26]. The genetic features associated with plasmid stability include, but are not limited to: machinery for self-transmissibility [27], insurance of a high copy number for dissemination to daughter cells during clonal expansion, avoidance of multimers [28] and the killing of host cells that do not contain a plasmid [29]. At present, it is not understood how the specific genes underpinning stability facilitate the ongoing maintenance of multidrug-resistant plasmids, especially as antibiotic usage changes [26]. This line of study was previously limited by the ability to attribute genes to plasmid or chromosomal origin.

Technological advances in recent years have significantly improved our ability to characterize plasmids, particularly the advances in the availability, cost and accuracy of long-read sequencing). This technology has become widespread and allows researchers to reconstruct complete plasmid genomes and examine their genetic architecture. The aim of this study was to provide a detailed comparison of the most common, circulating cefotaximase-harbouring plasmids in *E. coli* isolated from UK livestock and meat samples between the years 2013–2020 as part of the UK national monitoring programme for AMR. This will provide insight into the genetic factors that promote the maintenance of epidemic plasmids and elucidate how different incompatibility types/cefotaximase combinations have evolved over time. This information is critical to our understanding of the role of plasmids in persistence of ESBL *E. coli* in livestock and meat products and inform how this has changed with nationwide reductions in antimicrobial usage. It also demonstrates how genomic epidemiology can be used to assess the risk posed by epidemic plasmids harbouring critical AMRs.

## Methods

### Extraction of meat/caecal *E. coli* DNA

The national monitoring dataset comprised 1,379 ESC harbouring *E. coli* isolates of caecal or meat origin, collected between 2013 and 2020. The caecal samples were collected from abattoirs from animals that are entering the food chain and the meat samples were collected from supermarkets [7,19, 20, 25]. Most of the isolates recovered were of porcine (*n*=596), broiler (*n*=253) or chicken meat (*n*=409) origin (Table 1). *E. coli* from pig caeca in 2013 originated from archived isolates from a nationwide porcine study by Randall *et al*. 2014 [16] and were extracted as previously described [25]. All other isolates originated from *E. coli* collected as part of an AMR monitoring programme undertaken by the UK, where samples were collected from pigs and poultry on alternate years (pigs odd and poultry even). MICs to a panel of 16 antimicrobials were obtained as per EFSA guidelines, as previously described [25]. Isolates from 2015 were extracted from crude boilate as described in [30] and the remainder were extracted using the KingFisher Flex system (Thermo Fisher, USA) from 3 ml overnight culture in Lysogeny broth (LB) as per [25].

**Table 1.** The number of *E. coli* isolates collected in each year as part of the UK national livestock surveillance programme at the Animal and Plant Health Agency (APHA) between 2013 and 2020. Isolates were of porcine, poultry, cattle and lamb origin and were grown on selective ESC agar

Host animal/Meat type	Year							
	2013	2015	2016	2017	2018	2019	2020	Total
**Beef meat**	0	2	0	2	0	1	0	5
**Broiler caeca**	0	0	178	0	41	0	34	253
**Chicken meat**	0	0	241	0	73	0	95	409
**Lamb meat**	0	0	0	0	0	0	3	3
**Pig caeca**	185	186	0	127	0	98	0	596
**Pork meat**	0	6	0	1	0	7	0	14
**Turkey caeca**	0	0	26	0	17	0	7	50
**Turkey meat**	0	0	0	0	0	0	49	49
**Total result**	185	194	445	130	131	106	188	1,379

For Oxford Nanopore sequencing, a single colony grown on MacConkey-CTX agar was inoculated into 500 µl lysogeny broth without glucose (LB-G) and grown aerobically overnight at 37 °C and 150 r.p.m. Samples were pelleted at and washed with 0.9% saline and stored in DNA/RNA shield overnight at 4 °C. Samples were lysed after 16–18 h (‘QuickExtract’ Bacterial DNA Extraction Solution and ‘Ready-Lyse’ Epicentre) and treated with Proteinase-K (Qiagen). Finally, sodium acetate precipitation was used to purify the resulting product. The DNA was barcoded using the rapid-barcoding kit and ran on a R9.4 MinION flow cell.

### WGS and analysis

All isolates from 2013 were sequenced with Illumina HiSeq and with Illumina NextSeq for subsequent years. A subset of 43 samples were sequenced using Oxford Nanopore MinION Rapid-12 and Rapid-96 barcoding kit and R.9.4.1 flowcell (SQK-RBK110.96 and SQK-RBK004, Oxford Nanopore, UK).

SPAdes v3.11.1 was used for the assembly of Illumina reads and hybrid assemblies were generated using Unicycler v0.4.8. MLST of the bacterial host was assigned using SRST2 v0.2.0 [31] and AMR genes were found using APHA SeqFinder v4.0.6 [30,32] and Abricate v1.0.1 [33], yielding 1806 contigs that harboured a beta-lactamase gene. Where circularized, plasmid/CTX encoding contigs were extracted from hybrid assemblies and annotated using Bakta 1.11.0. ‘MOB_typer’ from MOBSUITE v3.1.9 [34] was used to investigate the host range and predicted mobility of each plasmid. k-mer-based genetic similarity was calculated with Sourmash v3.5.0 [35], with a k-mer size of 31. Based on the result of Sourmash, IncI1/*bla*_CTX-M-1_ and IncI1/*bla*_CTX-M-14_ plasmids were chosen to investigate the potential factors contributing to the high prevalence of IncI1/*bla*_CTX-M-1_ plasmids. SNIPPY v4.6.0 was used for variant calling with pPE13-080 as the reference sequence and snp-dists v0.8.2 for subsequent calculation of SNP distances between IncI1 plasmids [36,37]. Finally, core (present in all plasmids) and accessory genomes between circularized IncI1/*bla*_CTX-M-1_, IncI1/*bla*_CTX-M-14_ or IncF plasmids were analysed using Panaroo v1.6.0 with sensitive clean mode [38], before analysis of the gene presence absence using Scoary to establish statistical significance. The pangenome reference outputs from Panaroo were put through eggnog mapper v6.0.0 with database 5.0.2 to cluster genes by functions for input into Clinker v0.0.32 for multiple sequence alignment [39,40]. BRIG v0.95 was used for alignment and visualization of circular plasmid maps [41].

Finally, to identify the prevalence of IncI1/*bla*_CTX-M-1_ and IncI1/*bla*_CTX-M-14_ plasmids that are circulating among the *E. coli* isolates, a custom database of the fully circularized plasmids of both types (*n*=18) was created. blastn was used to find the top hits for these plasmids in the short-read assemblies identified as positive for bearing IncI1 plasmids (*n*=924) with an identity >=99%, a cumulative coverage of >=98% and a minimum length of 1,000 bp [42]. This method was validated in two ways: (i) it was verified that blastn at these thresholds could correctly identify plasmids in ‘self’ isolates and (ii) alignment was conducted to verify that the contig hits for the most common query plasmids covered large portions of the reference plasmid (see supplementary materials for details of method validation).

### Plasmid mobility

Four donor (D) IncI1/*bla*_CTX-M-1_ isolates (1796, PE13-80, 4619 and 5081) and two IncI1/*bla*_CTX-M-14_ isolates (4324 and 4636) and a recipient strain: J5-3 RifR (R1) were cultured aerobically from a single colony in 500 µl LB-G overnight at 150 r.p.m. and 37 °C with 1 mg ml^−1^ Cefotaxime for donor isolates and 125 µg ml^−1^ rifampicin for recipient isolates [43]. The following day, 100 µl of each was inoculated into 9,900 µl LB and allowed to reach 1×10^7^ c.f.u. ml^−1^. At this point, a mixed culture of a 1 : 1 ratio donor:recipient was made and incubated at 37 °C with no shaking. OD_600_ was measured every 5 min overnight for each culture using a Tecan automated plate reader at 37 °C overnight. At 2 h post-mating, the mating culture was plated on 1 mg ml^−1^ Cefotaxime+250 µg ml^−1^ rifampicin MacConkey agar in serial dilution to calculate the population density of the resulting transconjugants (T1). Positive and negative controls were also used to verify the selective criteria for the plates and to check for mutants. Transconjugants were collected and stored at −80 °C in glycerol before being used as donors (D2) for a repeat mating experiment, this time with a J5-3 AzR recipient strain (R2) to form transconjugants resistant to Cefotaxime and sodium azide (T2). If transconjugants (T1) grew for the second time on Cefotaxime+Rifampicin double-selective plates, they were accepted as transconjugants and not mutants. Where T2 grew on 250 µg ml^−1^ sodium azide +1 mg ml^−1^ cefotaxime double selective plates, the conjugation rate was calculated using the package ‘conjugator’ in R. Within the ‘conjugator’ package, the approximate Simonsen model (ASM), the Simonsen model and the ratio of transconjugants/donors×recipients (T/DR) were all used to calculate conjugation rate [30,44].

## Results

### The national monitoring dataset and *bla*-_CTX-M_ plasmid variants

**Fig. 1.**
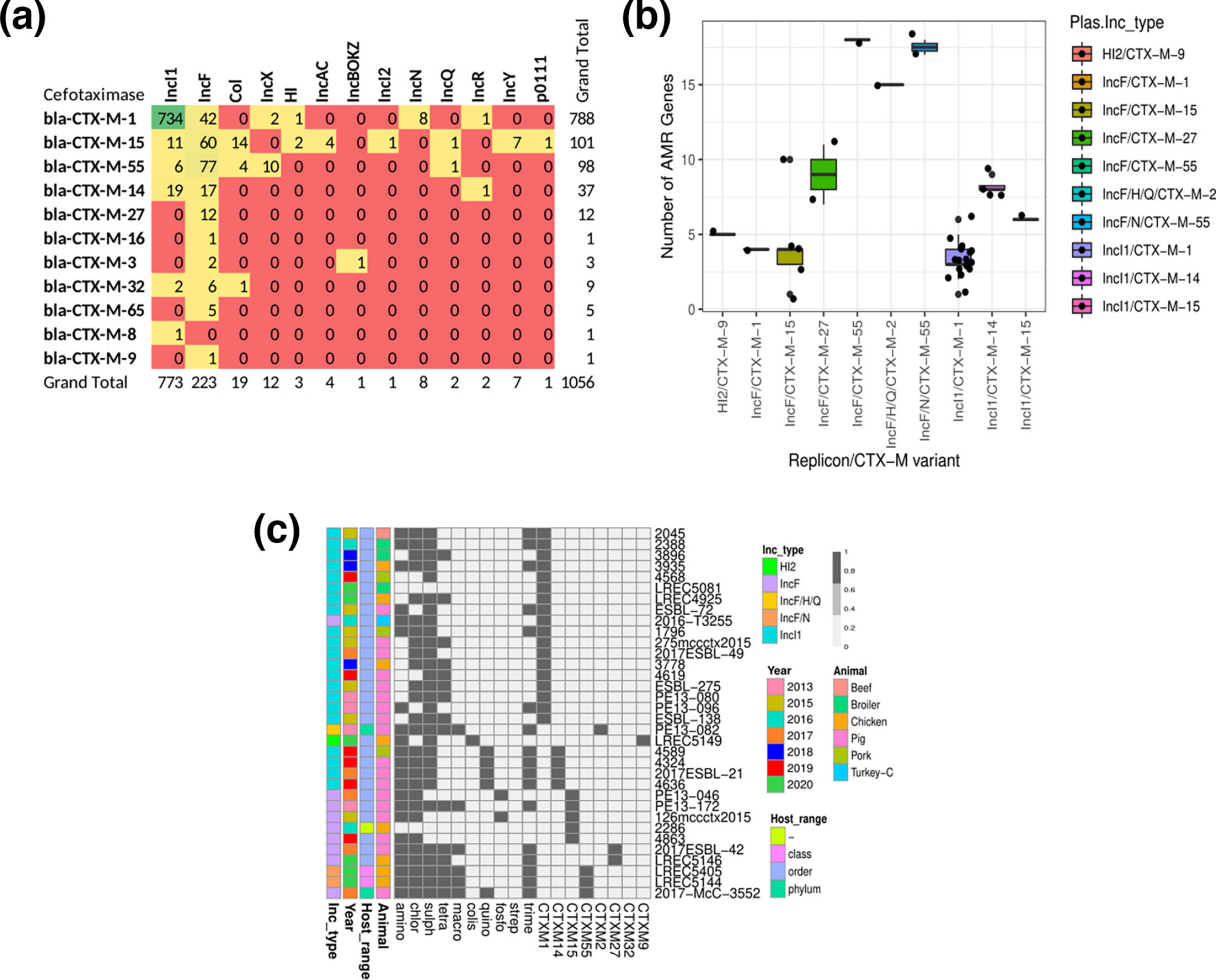
(**a**) A co-occurrence heatmap, showing the prevalence of cefotaximases among plasmid Inc types. (**b**) A boxplot showing the median number of AMR genes among plasmid Inc type/cefotaximase combinations. (**c**) A heatmap displaying the co-resistances harboured by different plasmid Inc types. Metadata pertaining to the isolate from which the plasmid was derived is encoded by colours in the key (right).

Illumina short-read WGS was performed on 1379 ESC *E. coli* collected through national surveillance of AMR in livestock in the UK, to investigate the most common plasmid incompatibility types present in isolates and their cefotaximases. In silico sequence typing revealed that 195 different *E. coli* STs were represented in the dataset, belonging to 31 ST clonal complexes. In the total isolate dataset, IncF plasmids were the predominant plasmid type, present in 83.9% of isolates, followed by IncI1 (67%), col (65.92%) and IncX (17.55%) (Table S1i and ii, available in the online Supplementary Material). There were 1,806 contigs containing beta-lactamase genes (*bla*_CTX-M_; *bla*_TEM_; *bla*_SHV_; *bla*_CMY;_*bla*_OXA;_*bla*_DHA;_*bla*_LAP-2_) and those with *ampC* promoter mutations. Approximately 60% (1,076/1,806) of all beta-lactamase containing contigs harboured at least one variant of *bla*_CTX-M._*bla*_CTX-M-1_ was the most common cefotaximase, present in 57.14% of all isolates, followed by *bla*_CTX-M-15_ (7.69%), *bla*_CTX-M-55_ (7.25%) and *bla*_CTX-M-14_ (2.68%; Table S2i). TEM, CMY and SHV were the next most prevalent beta-lactamases, with fewer contigs containing *ampC* promoter mutations, OXA, DHA and LAP-2 beta-lactamases (Table S2ii). APHA Seqfinder results showed that the most commonly co-occurring cefotaximase and Inc-type was IncI1/*bla*_CTX-M-1,_ which co-occurred in 734 isolates and, although IncF was a common replicon, it only co-occurred with CTX-M in 223 isolates and rarely on the same contig (Fig. 1a). The IncI1/blaCTX-M-1 combination was repeatedly identified within the same ST across multiple years and animal sources, most commonly ST602 (*n*=51), detected in six sources between 2013 and 2020 (Table S2iii). The prevalence of individual cefotaximases varied by year and animal (Tables 1 and S2iv).

Of the 43 isolates that were long-read sequenced, 35 contained plasmid replicons on the same contig as a *bla*_CTX-M_ and the remaining ten appeared to have chromosomal *bla*_CTX-M_. Isolates were selected based on diversity of their Inc types harbouring *bla*_CTX-M_ and the plasmids belonged to the following replicon types: IncI1 (*n*=22), IncF (*n*=9), HI2 (*n*=1), or multi-replicon IncF/N (*n*=2) and IncF/H/Q (*n*=1). Of the 35 that contained plasmid replicons on the same contig as cefotaximase, 29 of these were closed during assembly. The mean size of circularized IncI1 and IncF plasmids was 108,727 bp (range=92,223 : 118,642 bp) and 156,799 bp (range=67,881 : 170,866 bp), respectively (Table 2).

**Table 2.** The cefotaximase harbouring contigs and plasmids selected for ONT sequencing. Where plasmid replicons are present, the host range and level of predicted conjugative ability have been predicted using MOB suite. pMLST has been retrieved where possible for plasmids and metadata about the host bacterial strain, including MLST, animal/source and year is included

Strain ID	Plasmid type	Inc type	*bla*_CTX-M_	Circularized	Size (kb)	Conjugative	Host range	Animal	Year	Source	MLST	pMLST (IncI1)	Accession
LREC5150	None/Chr	–	CTX-M-1	No	51	Conjugative	Family	Chicken	2020	Meat	23	–	PX236809
4600	None/Chr	–	CTX-M-1	Yes	468	Non-mobilizable	–	Pig	2019	Caecal	101	–	JBREGE000000000
4557	None/Chr	–	CTX-M-14	No	301	Non-mobilizable	–	Beef	2019	Meat	117	–	JBREGD000000000
3908	None/Chr	–	CTX-M-14	Yes	478	Non-mobilizable	–	Turkey	2018	Caecal	93	–	JBREGF000000000
4663	None/Chr	–	CTX-M-15	No	20	Non-mobilizable	–	Pig	2019	Caecal	48	–	PX236808
LREC5338	None/Chr	–	CTX-M-15	Yes	318	Non-mobilizable	–	Turkey	2020	Meat	1163	–	JBREGC000000000
2017ESBL-342	None/Chr	–	CTX-M-32	No	307	Non-mobilizable	–	Pig	2017	Caecal	710	–	JBREGH000000000
LREC5384	None/Chr	–	CTX-M-55	Yes	14	Non-mobilizable	–	Chicken	2020	Meat	752	–	PX236810
2824	None/Chr	–	CTX-M-55	No	481	Non-mobilizable	–	Broiler	2016	Caecal	3776	–	JBREGG000000000
LREC5149	HI2, HI2A	HI2	CTX-M-9	Yes	249	Conjugative	Order	Chicken	2020	Meat	69	–	PV686675
2286	FIB X 2, FII, FIA	IncF	CTX-M-15	Yes	111	Non-mobilizable	Family	Chicken	2016	Meat	1594	–	PV686658
PE13-172	FIC X 2, FIA, FIB	IncF	CTX-M-15	Yes	170	Conjugative	Family	Pig	2013	Caecal	44	–	PV686681
PE13-046	FII	IncF	CTX-M-15	Yes	71	Conjugative	Family	Pig	2013	Caecal	710	–	PV686677
126mccctx2015	FII	IncF	CTX-M-15	Yes	72	Conjugative	Family	Pig	2015	Caecal	2064	–	PV686651
4863	FII	IncF	CTX-M-15	Yes	67	Conjugative	Family	Pig	2019	Caecal	88	–	PV686670
LREC5146	FIB X2, FII, FIA	IncF	CTX-M-27	Yes	121	Conjugative	Family	Chicken	2020	Meat	533	–	PV686674
2017ESBL-42	FIC, FIA, FII, FIB	IncF	CTX-M-27	Yes	134	Conjugative	Family	Pig	2017	Caecal	131	–	PV686655
2017-McC-3552	FIA/FIB/FII	IncF	CTX-M-55	No	201	Conjugative	Family	Pig	2017	Caecal	101	–	PV686687
PE13-082	IncQ/HI1B/HI1A/FIA	IncF/H/Q	CTX-M-2	No	227	Conjugative	Family	Pig	2013	Caecal	93	–	PV686679
LREC5405	FIB X2/IncN/FIC	IncF/N	CTX-M-55	Yes	140	Conjugative	Family	Chicken	2020	Meat	752	–	PV686676
LREC5144	FIB/FIC/IncN	IncF/N	CTX-M-55	Yes	232	Conjugative	Genus	Chicken	2020	Meat	752	–	PV686673
2016-T3255	IncI1	IncI1	CTX-M-1	No	104	Conjugative	Order	Turkey	2016	Caecal	410	3	PV686653
ESBL-72	IncI1	IncI1	CTX-M-1	No	89	Conjugative	Order	Pig	2015	Caecal	847	–	PV686688
3896	IncI1	IncI1	CTX-M-1	Yes	110	Conjugative	Order	Broiler	2018	Caecal	57	–	PV686662
3935	IncI1	IncI1	CTX-M-1	Yes	113	Conjugative	Order	Chicken	2018	Meat	7013	–	PV686663
4568	IncI1	IncI1	CTX-M-1	Yes	100	Conjugative	Order	Pork	2019	Meat	101	–	PV686665
LREC5081	IncI1	IncI1	CTX-M-1	Yes	92	Conjugative	Order	Broiler	2020	Caecal	1611	58	PV686672
LREC4925	IncI1	IncI1	CTX-M-1	Yes	104	Conjugative	Order	Chicken	2020	Meat	10	3	PV686671
1796	IncI1	IncI1	CTX-M-1	Yes	108	Conjugative	Order	Pork	2015	Meat	602	3	PV686652
275mccctx2015	IncI1	IncI1	CTX-M-1	Yes	107	Conjugative	Order	Pig	2015	Caecal	2144	3	PV686660
2017ESBL-49	IncI1	IncI1	CTX-M-1	Yes	105	Conjugative	Order	Pig	2017	Caecal	1582	3	PV686656
3778	IncI1	IncI1	CTX-M-1	Yes	105	Conjugative	Order	Chicken	2018	Meat	1485	3	PV686661
4619	IncI1	IncI1	CTX-M-1	Yes	110	Conjugative	Order	Pig	2019	Caecal	101	3	PV686666
PE13-080	IncI1	IncI1	CTX-M-1	Yes	107	Conjugative	Order	Pig	2013	Caecal	23	3	PV686678
PE13-096	IncI1	IncI1	CTX-M-1	Yes	110	Conjugative	Order	Pig	2013	Caecal	453	–	PV686680
ESBL-138	IncI1	IncI1	CTX-M-1	Yes	108	Conjugative	Order	Pig	2015	Caecal	155	3	PV686668
2045	IncI1	IncI1	CTX-M-1	No	84	Conjugative	Order	Beef	2015	Meat	602	–	PV686657
2388	IncI1	IncI1	CTX-M-1	Yes	108	Conjugative	Order	Broiler	2016	Caecal	602	3	PV686659
4589	IncI1	IncI1	CTX-M-14	Yes	114	Conjugative	Order	Pork	2019	Meat	10	–	PV686669
4324	IncI1	IncI1	CTX-M-14	Yes	118	Conjugative	Order	Pig	2019	Caecal	369	80	PV686664
2017ESBL-21	IncI1	IncI1	CTX-M-14	Yes	117	Conjugative	Order	Pig	2017	Caecal	278	80	PV686654
4636	IncI1	IncI1	CTX-M-14	Yes	118	Conjugative	Order	Pig	2019	Caecal	10	80	PV686667
PE13-071	IncI1	IncI1	CTX-M-15	No	87	Conjugative	Order	Pig	2013	Caecal	6158	31	PV686689

### Comparative genomics of CTX harbouring plasmids

**Fig. 2.**
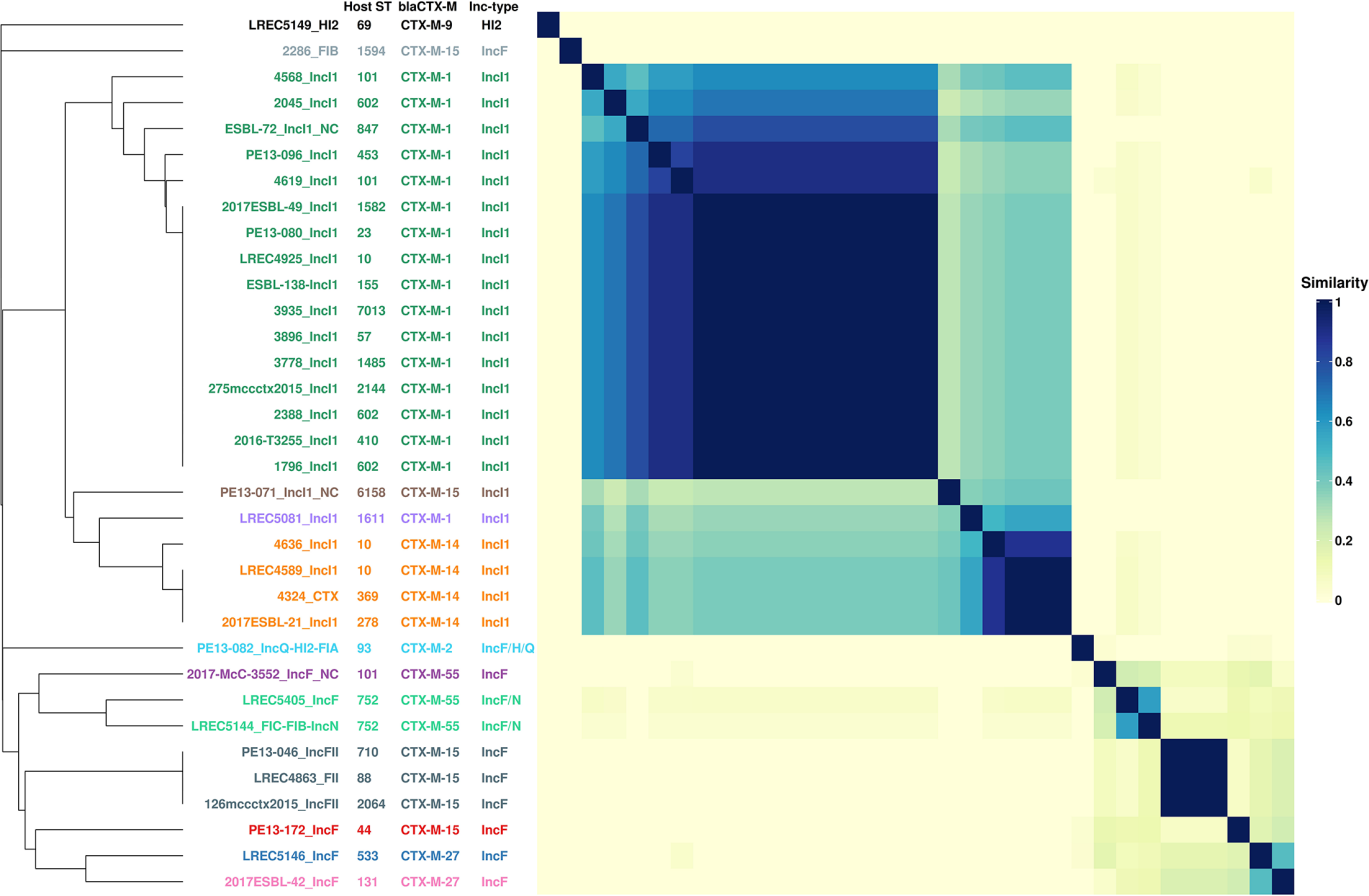
k-mer-based homology using Sourmash of all circularized plasmids that harboured bla-_CTX-M_ variants from samples chosen for SMRT sequencing and subsequent hybrid assembly. Text colour signifies clade membership, with *E. coli* host ST, *bla*_CTX-M_ variant and Inc-type information also provided.

**Fig. 3.**
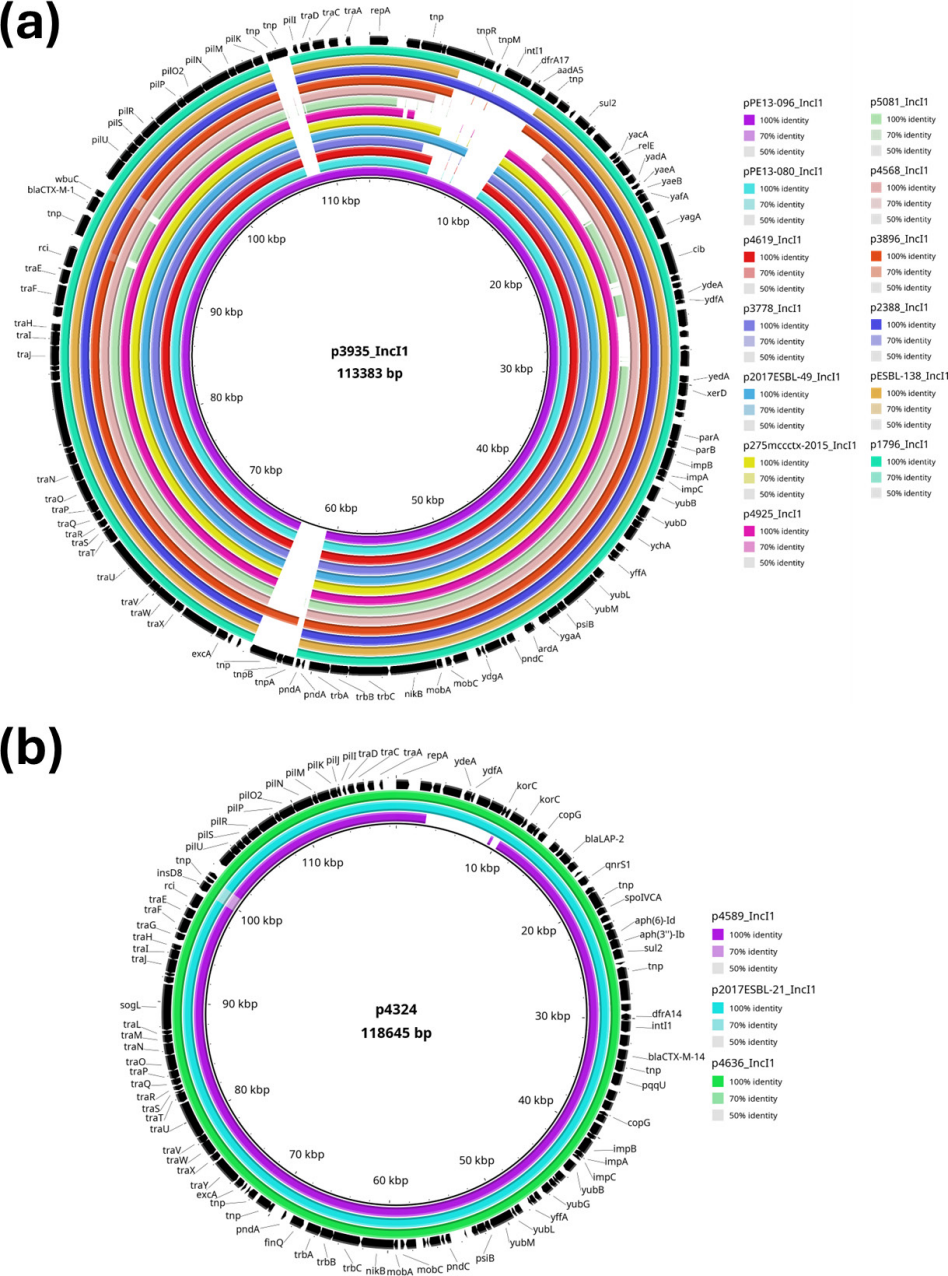
BRIG annotation visualizations of (**a**) IncI1/CTX-M-1 plasmids (*n*=14) and (**b**) IncI1/CTX-M-14 plasmids (*n*=4). The outer ring is the annotation for the reference plasmid, p3935, depicted in black.

APHA SeqFinder output was used to examine which AMR genes were present in the long-read isolates and whether they were associated with specific plasmid types to understand how each resistance profile may circulate among bacterial hosts. The Inc type/*bla*_CTX-M_ variant combination with the highest mean number of AMR genes was IncF/N/*bla*_CTX-M-55_ (17.5±0.50). IncI1/*bla_CTX-M-1_* plasmids had a lower average number of AMR genes (3.5±0.28) compared with IncI1/*bla*_CTX-M-14_ plasmids (8.25±0.25) (Fig. 1a, b). All the IncI1/*bla*_CTX-M-14_ co-harboured the same resistances (trimethoprim, ciprofloxacin, sulphamethoxazole, chloramphenicol and aminoglycoside), although IncI1/*bla*_CTX-M-1_ and IncF/*bla*_CTX-M-15_ plasmids varied to a greater extent in their relative to one another (Fig. 1c). Most of the long-read IncF plasmids characterized had the predicted host range of Enterobacteriaceae, although one was predicted to have a host range of only *Escherichia*. In contrast, the IncI1 all had the predicted host range of Enterobacterales (Table 2).

k-mer-based homology performed using Sourmash highlighted that within IncI1 plasmids, those encoding *bla*_CTX-M-1_ shared between 60–100% k-mer sequence similarity (dark green clade, Fig. 2), and some showed >70% similarity, forming a separate sub-cluster on a plasmid genome-based phylogenetic tree. Furthermore, IncI1 plasmids harbouring *bla*_CTX-M-14_ (orange clade) shared >70% similarity and separated into a different sub-cluster, as did the IncFII plasmid subcluster with *bla*_CTX-M-15_ (dark grey clade). Comparison of plasmid genomes indicated that all IncI1/*bla*_CTX-M-1_ and IncI1/*bla*_CTX-M-14_ plasmids harboured genes relating to addiction systems, dimer avoidance, SOS response, substrate metabolism and toxins against Gram-negative bacteria. Insertion sequence (IS) element enrichment was also a key feature of both plasmid types (Fig. 3).

**Fig. 4.**
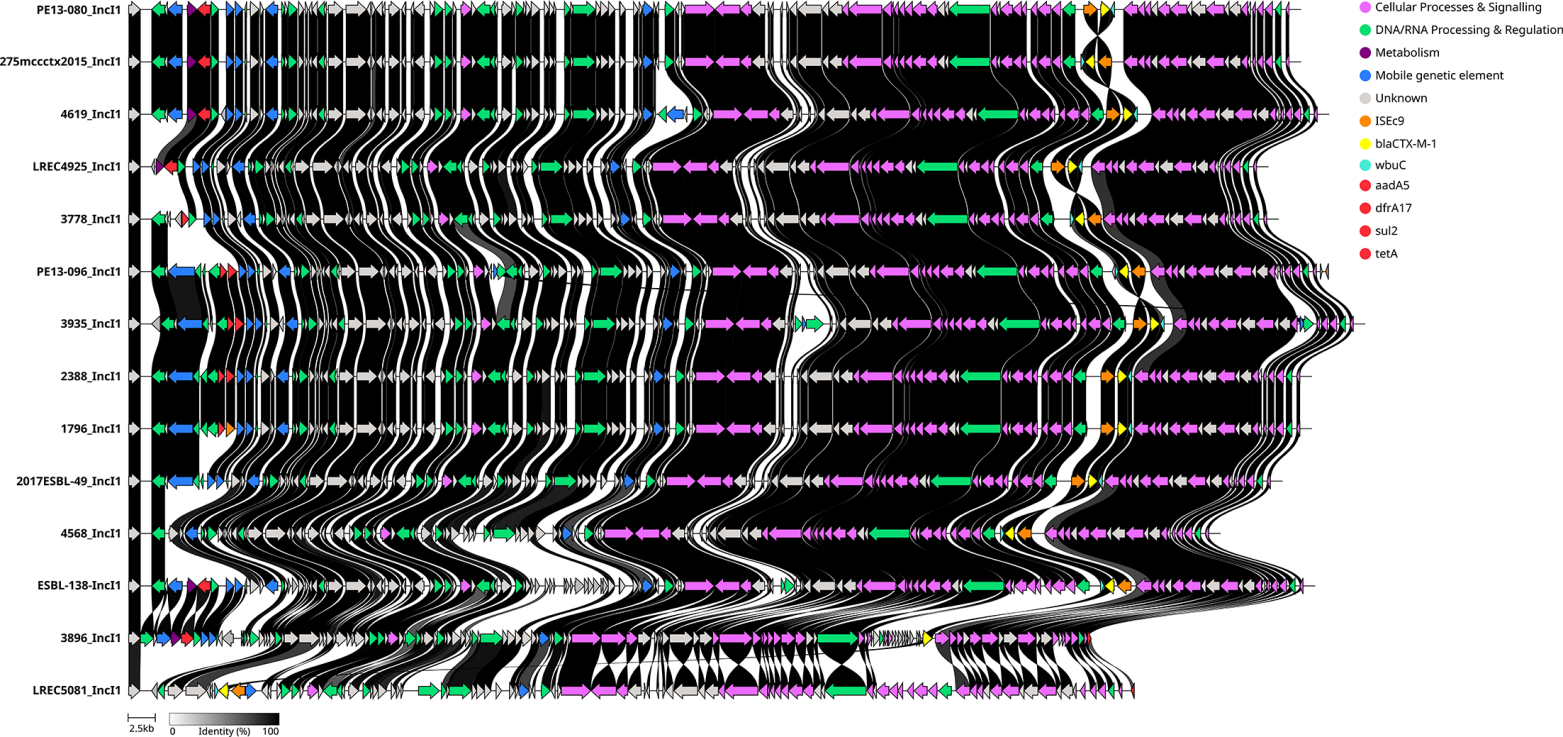
Clinker alignment of circularized IncI1/*bla*_CTX-M-1_ plasmids with coloured key genes and clustered gene functions. Similarity between genes is indicated by darker lines and gaps indicate genes not shared between plasmids.

**Fig. 5.**
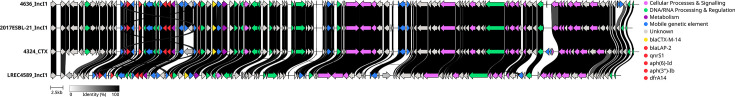
Clinker alignment of circularized IncI1/*bla*_CTX-M-14_ plasmids with coloured key genes and clustered gene functions. Similarity between genes is indicated by darker lines and gaps indicate genes not shared between plasmids.

In the circularized plasmid genome, there were three distinct ‘hotspots’ of variation identified when all IncI1/*bla*_CTX-M-1_ plasmids were compared using BRIG to the circularized reference genome (Fig. 3); they were at 5–10 kb, 60–65 kb, 110 kb downstream of the *rep2* gene. There was also with some variation in genes flanking *bla*_CTX-M-1_ in some plasmids. The 5–10 kb variable hotspot showed gene differences mostly corresponding to presence/absence of IS4 transposase, a class I integron *intI1* and *aadA5*, usually present in class I integrons. In the 60–65 kb region, p3935 and p3778 shared a similar gene block harbouring a mobile element protein, which was absent in the remaining plasmid genomes. *E. coli* isolates harbouring these plasmids were both isolated from the same year but from a retail chicken meat and a broiler caecum. In most IncI1/*bla*_CTX-M-1_ plasmids, the *ISEc9-bla_CTX-M-1_-wbuC* cassette lies between 85 and 95 kb. However, this region was inverted in p275mccctx2015, pPE13-096, pESBL-1384568, p5081 and p3778 (Fig. 4). In p5081, the inverted *ISEc9-bla_CTX-M-1_-wbuC* cassette fell at around 9 kb. This marked difference in p5081 may be in part due to it belonging to a different lineage than the other IncI1/*bla*_CTX-M-1_ plasmids, as demonstrated by the pMLST class and clustering (Table 2; Fig. 2). Perhaps due to their limited sample size, the IncI1/*bla*_CTX-M-14_ plasmids included in this study were far more conserved with minimal variation, and all exhibited common features. Although there were some differences, such as inversion of mobile genetic elements adjacent to the region that harboured multiple AMR genes. p4589 was missing the gene *ibfA* between *repA* and *korC* at the 3 kb-10 kb region relative to the other plasmids (Figs 5 and S1).

When circularized IncI1/*bla*_CTX-M-1_(*n*=14) and IncI1/*bla*_CTX-M-14_ (*n*=4) plasmid genomes were compared, 65/235 genes were common to both plasmid types. These genes included functions involved in conjugative transfer, pilus biogenesis, plasmid maintenance, DNA replication and a group of hypotheticals. We identified 13 genes unique to all IncI1/*bla*_CTX-M-1_ plasmids and 39 genes unique to all IncI1/*bla*_CTX-M-14_ plasmids. However, due to the small sample size, none of the genes were deemed significant to either group when considering the Bonferroni corrected *P*-values. In contrast to the IncI1, the circularized IncF (*n*=9) plasmids shared no core genes out of the 516 identified. There were 10 genes that were shared amongst eight of the plasmids, with only 2286_FIB not harbouring them, which matched the clustering of the IncF plasmids (Fig. 2). These genes included those for plasmid maintenance (repA-FII for replicon initiation, pemIK for toxin-antitoxin), DNA modification and some with poor characterization. All of them were significantly associated with IncF plasmids in addition to a further seven genes that were not identified in the IncI1 group (Bonferroni *P*=0.0027). Conversely, there were a group of 65 significantly associated (Bonferroni *P*=0.00014–0.0078) genes only identified in the IncI1 plasmids, the majority of which were in the IncI1 core (55/65).

Isolates 3908 and 4557 both harboured *bla*_CTX-M-14_ but not in association with a plasmid replicon, with genome assemblies suggesting chromosomal location. Core genome analysis showed that IncI1/*bla*_CTX-M-14_ plasmids share only two genes with 3908 and 4557: a hypothetical protein and IS family transposase IS30. This suggested integration of *bla*_CTX-M-14_ via a transposable element in the chromosome of these isolates.

Screening presence for both plasmid types in the 924 *E. coli* harbouring IncI1 using blastn against a customized database of our circularized resolved plasmids revealed that 911/924 (98.6%) of isolates verified to be harbouring an IncI1 plasmid, harboured a plasmid type with high sequence similarity to plasmids in either the green clade or the orange clade of the sourmash distance tree (Fig. 2). Of these, 734 were IncI1/*bla*_CTX-M-1_ and just 19 were IncI1/*bla*_CTX-M-14_. IncI1/*bla*_CTX-M-1_ were most prevalent in isolates from pig caeca, followed by chicken meat and then broiler caeca (Table 3a). They were least prevalent in pork. Samples were taken on alternate years for pigs and poultry but overall, the prevalence of IncI1/*bla*_CTX-M-1_ decreased over time in porcine from 115 isolated in 2013 from pig caeca to just 51 from pig caeca in 2019. The prevalence of IncI1/*bla*_CTX-M-1_ was initially very high in poultry samples and, as seen with porcine, decreased over time between 2016 and 2020. The prevalence of IncI1/*bla*_CTX-M-1_ was, however, highest in isolates from chicken meat, relative to broiler caeca or turkey caeca and was completely absent in turkey meat throughout the period sampled.

**Table 3.** (a) Prevalence of the IncI1/bla-CTX-M-1 and IncI1/bla-CTX-M-14 plasmids that were used as a custom database for blastn by year and animal/meat the bacteria was isolated from. (b) The five plasmids with the highest number of hits in the short-read assemblies verified to harbour an IncI1 plasmid according to APHA-SeqFinder (*n*=924)

	**Year**								
**(a)**	**2013**		**2015**	**2016**	**2017**	**2018**	**2019**	**2020**	**Grand total**
**Beef**	**0**		**1**	**0**	**1**	**0**	**0**	**0**	**2**
IncI1 CTX-M-1	0		1	0	1	0	0	0	2
**Chicken caeca**	**0**		**0**	**112**	**0**	**18**	**0**	**8**	**138**
IncI1 CTX-M-1	0		0	112	0	18	0	8	138
**Chicken meat**	**0**		**0**	**171**	**0**	**51**	**0**	**15**	**237**
IncI1 CTX-M-1	0		0	171	0	51	0	15	237
IncI1 CTX-M-14	0		0	0	0	0	0	1	1
**Pig**	**117**		**106**	**0**	**66**	**0**	**53**	**0**	**342**
IncI1 CTX-M-1	115		100	0	62	0	48	0	325
IncI1 CTX-M-14	2		6	0	4	0	5	0	17
**Pork**	**0**		**5**	**0**	**0**	**0**	**4**	**0**	**9**
IncI1 CTX-M-1	0		5	0	0	0	3	0	8
IncI1 CTX-M-14	0		0	0	0	0	1	0	1
**Turkey caeca**	**0**		**0**	**16**	**0**	**6**	**0**	**1**	**23**
IncI1 CTX-M-1	0		0	16	0	6	0	1	23
**Turkey meat**	**0**		**0**	**0**	**0**	**0**	**0**	**1**	**1**
IncI1 CTX-M-1	0		0	0	0	0	0	1	1
**Grand total**	**117**		**112**	**299**	**67**	**75**	**57**	**25**	**753**
**(b) Plasmid ID**	**Year of isolate collection**		**Resistances carried by plasmid**				**Size/bp**	**No. of isolates with 98% identity**	
**p2388**	2016		Trimethoprim, Sulphamethoxazole, Aminoglycoside, Beta-lactams				108,489	177	
**pLREC4925**	2020		Tetracycline, Sulphamethoxazole, Beta-lactams				104,515	155	
**pPE13-80**	2015		Tetracycline, Trimethoprim, Sulphamethoxazole, Aminoglycoside, Beta-lactams				107,495	115	
**p3778**	2013		Tetracycline, Sulphamethoxazole, Beta-lactam				105,451	111	
**p1796**	2018		Trimethoprim, Sulphamethoxazole, Beta-lactams				108,489	98	
**(c)**	**p2388**	**pLREC4925**	**p1796**	**p3778**		**pPE13-80**			
**p2388**	0	1	3	1		14			
**pLREC4925**	1	0	4	0		16			
**p1796**	3	4	0	4		17			
**p3778**	1	0	4	0		15			
**pPE13-80**	14	16	17	17		0			

**Fig. 6.**
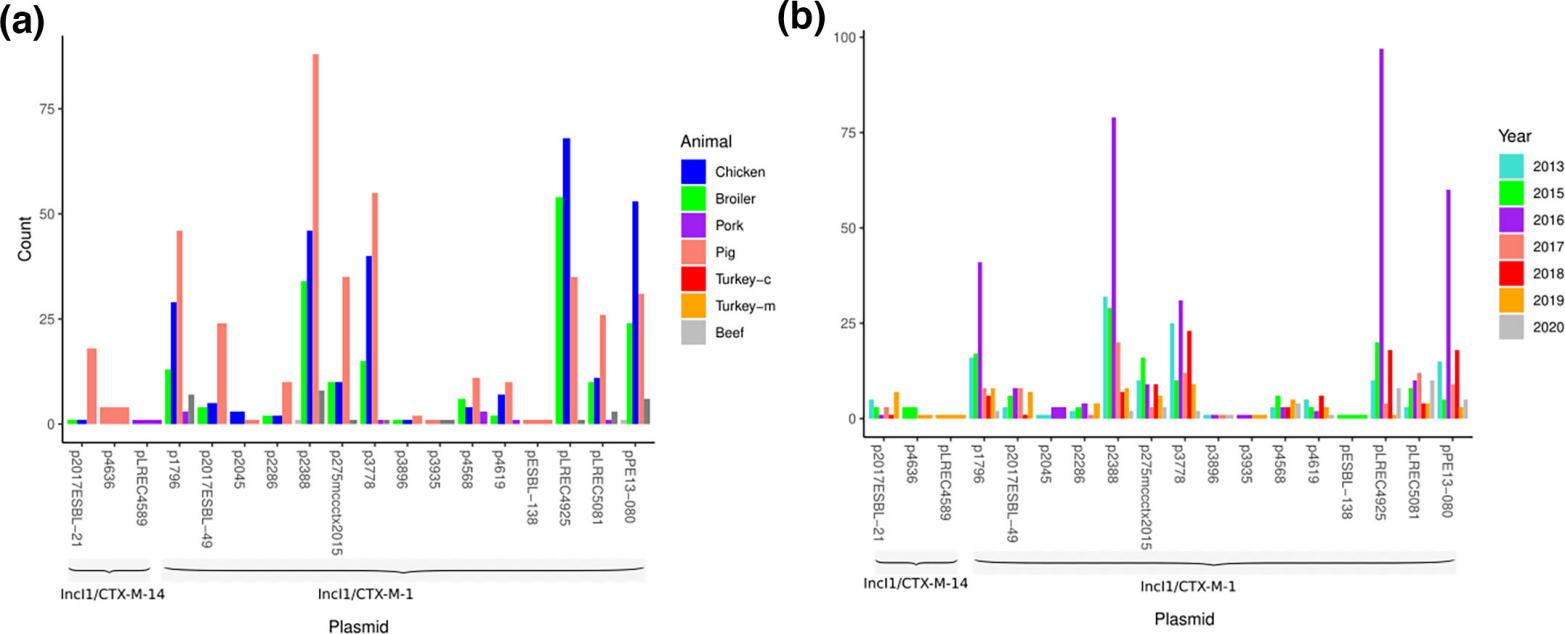
The count of isolates with the best hit matching circularized IncI1/*bla*_CTX-M-14_ or IncI1/*bla*_CTX-M-1_ plasmids by (**a**) animal/meat and (**b**) year. The best hit was defined as the hit with the first best coverage when a database of the circularized plasmid assemblies was used to screen short reads for hits matching the plasmids with >98% identity, >1,000 bp length and >50% cumulative coverage. Chicken and turkey-m refer to meat and broiler and turkey-c to caeca.

The plasmids with the greatest number of hits according to the thresholds specified in the methods were all IncI1/*bla*_CTX-M-1_ plasmids, isolated from *E. coli* from AMR monitoring spanning 2013–2020 (Fig. 6; Table 3b). The plasmids, in addition to cefotaxime, encoded co-resistances to trimethoprim, aminoglycoside and sulphamethoxazole, or tetracycline, aminoglycoside and sulphamethoxazole, or both of the aforementioned groups of resistances. Furthermore, the SNP differences between the resolved circularized IncI1/*bla*_CTX-M-1_ plasmids was <20, when pPE13-80 was used as a reference due to its early sampling year (2013) (Table 3c). The plasmids that were characterized were entirely absent in isolates from turkey meat and had the highest prevalence in isolates from pig caeca, chicken meat and broiler caeca. IncI1/*bla*_CTX-M-1_ plasmids p1796, pLREC4925, p2388 and p3778 were the most prevalent in pigs and these plasmids were also the most prevalent in poultry, along with pPE13-080 and p275mccctx2015. Due to the high number of ESBL isolates originating from poultry in 2016, these plasmids appeared to be most prevalent in 2016 relative to the other sampling years.

### Mobilization of IncI1/*bla*_CTX-M-1_ versus IncI1/*bla*_CTX-M-14_ plasmids

Four IncI1/*bla*_CTX-M-1_ plasmids (p1796, pPE13-80, p4619 and p5081) and two IncI1/*bla*_CTX-M-14_ plasmids (p4324 and p4636) were chosen as representatives based on the BRIG data and predicted conjugative status (Table 2) and tested for mobility in liquid-mating culture. Of the IncI1/*bla*_CTX-M-1_ plasmids, p5081 and pPE13-80 were mobilizable, with estimated conjugation rates of 1e-09 for p5081 and 1e-9.5 for pPE13-080. Both IncI1/*bla*_CTX-M-14_ plasmids were also mobilizable with an estimated conjugation rate of 1e-09 for p4324 and p4636.

## Discussion

Cefotaximases belong to five groups (group 1, 2, 8, 9 and 25) with group 1 cefotaximases (*bla*_CTX-M-1_, _-15_, _-3_, _-55_, _-32_) and group 9 (*bla*_CTX-M-14_, _-9_, _-27_) being most widespread in Europe and UK [25,45]. Indeed, group 1 (*bla*_CTX-M-1_, *bla*_CTX-M-55_ and *bla*_CTX-M-15_) and group 9 (*bla*_CTX-M-14_) were the predominant cefotaximases in our data. Our ability to predict the likelihood of finding each cefotaximase/plasmid was limited due to the sampling of different animals/meats on alternate years, which created an interaction between species and year. However, in pigs and poultry at least (the most numerous sample types), there was an overwhelming predominance of *bla*_CTX-M-1_ from 2013 to 2020 (>55% in broilers, chickens and pigs). However, more recent UK surveys of poultry caeca and meat have found other ESC mechanisms are becoming more common [20]. The incompatibility groups IncF, IncI1, Col and IncX were the most common plasmid types and IncI1/*bla*_CTX-M-1_ was a common combination. This is similar to the results obtained from clinical *E. coli* isolated in Ireland in 2009–2010, where IncF and IncI1 plasmids were among the most common incompatibility groups to harbour cefotaximases [46]. This study focused on IncI1 as it was the largest group and IncF has been studied previously with more variability [32].

k-mer-based homology highlighted that there is genetic similarity within plasmids Inc/CTX-M groups and, from the samples chosen here, there was no obvious clustering by animal host species, source or year. Notably, IncI1/*bla*_CTX-M-1_, IncI1/*bla*_CTX-M-14_ and IncFI/*bla*_CTX-M-15_ exhibited high similarity within their respective groups. Coupled with the high co-occurrence of IncI1 and *bla*_CTX-M-1_ in our data, this indicated that (i) IncI1/*bla*_CTX-M-1_ plasmids have remained stable over time and in different compartments (meat/caeca) and (ii) these may be ‘epidemic’ plasmids. Detailed genetic comparison between the 14 IncI1/*bla*_CTX-M-1_ and four IncI1/*bla*_CTX-M-14_ plasmids indicated that several genes may underpin the relative stability of IncI1/*bla*_CTX-M-1_. The bacteriocin encoding gene, *cia* (colicin -la) was present on all the IncI1/*bla*_CTX-M-1_ plasmids but on none of the IncI1/*bla*_CTX-M-14_ plasmids, which were less prevalent in our dataset. Two studies on *E. coli* isolated from human clinical samples (2017) [47] and from mastitic milk and cattle faecal samples (2004–2010) in France and Germany [48] have also recorded the presence of *cia* in IncI1/*bla*_CTX-M-1_ plasmids, indicating that the bacteriocin may provide a competitive advantage through competition exclusion and thus, a higher likelihood of survival in the gastrointestinal tract. Other genes involved with sugar metabolism (*glmM*), recombinational DNA repair (*dinG*) and the promotion of recombination (*rnpA_2*) also distinguished IncI1/*bla*_CTX-M-1_ from IncI1/*bla*_CTX-M-14_ plasmids. These may permit the metabolism of different substrates, allow DNA repair following damage and promote the movement of adaptive gene cassettes, which may all aid in the survival of the bacteria in livestock hosts. However, it should be reiterated that this comparison was limited by the number of circularized plasmids available within each of the groups and a wider sample size would be required to establish genes that are statistically significant.

Several results from this study are in line with previous findings in isolates from UK livestock. Results here suggest that the plasmids vary in their co-resistances according to Inc-type, which is concurrent with findings by Kirchner *et al*. [49]. In addition, results demonstrate that IncI1 plasmids tend to have fewer non-cefotaximase AMR genes relative to other Inc types, like the multireplicon IncF plasmids presented here. However, the present study demonstrates also that co-resistances also vary by the cefotaximase present, as evidenced by the same AMR profile in IncI1/*bla*_CTX-M-14_ plasmids and the similarity in IncI1/*bla*_CTX-M-1_ plasmids which always also encoded *sul2*, often with the addition of *tet(A*) or *aadA5, tet(A*) and *dfr(A*). Sulphonamide, tetracycline, streptomycin and trimethoprim are all important antimicrobials in veterinary use and, therefore, resistance to these would be advantageous to *E. coli* in livestock. The ability to mobilize such genes may, therefore, be advantageous to the bacterial host despite any energetic costs they may incur to do so.

Many of the cefotaximase harbouring plasmids in this study were predicted as ‘conjugative’, meaning that they have all the machinery necessary for dissemination via conjugation and represent a risk for horizontal gene transfer. Most of the host range predicted was confined to the order Enterobacterales, but concerningly, the multireplicon plasmid harbouring X/Y Inc-types p2017-McC-3552 had a host range spanning phylum, as well as bearing the greatest number of co-resistances. Conjugation experiments on a subset of IncI1/*bla*_CTX-M-1_ and IncI1/*bla*_CTX-M-14_ plasmids showed that all the *bla*_CTX-M-14_ plasmids are indeed mobilizable, but only 2/4 of the *bla*_CTX-M-1_ plasmids tested moved across to recipient isolates under our laboratory conditions. This may have been due to experimental conditions being unfavourable for those isolates to conjugate, or because the experiment did not run for a period long enough for conjugative pili formation. The conjugative potential of IncI1/*bla*_CTX-M-1_ did not seem very different from IncI1/*bla*_CTX-M-14_, although it was difficult to assess whether, overall, *bla*_CTX-M-1_ harbouring plasmids were more conjugative than those harbouring *bla*_CTX-M-14_ from the sample size tested here. The conditions we used for conjugation were not exhaustive so future work could include mimicking the real-world conditions of the isolates harbouring plasmids with the use of *in vitro* gut models [50].

Genes encoding CTX-M enzymes often lie in multidrug resistance regions (MRRs) downstream of ISs, such as ISEcp1 or ISCR1, which promote the spread of ARGs on ESBL plasmids [51]. Another IS thought to be heavily involved in the spread of *bla*_CTX-M_ is IS26 which is also often found on MRRs [52]. ISs can facilitate the genetic rearrangement of MRRs between ESBL plasmids, generating myriad host ranges and new resistances, e.g. to carbapenems [53]. In most of the IncI1/*bla*_CTX-M-1_ plasmids, the ISEcp9- *bla*_CTX-M-1_-*wbuC* cassette was conserved and lay between 85 and 95 kb on the resolved plasmid genomes. However, this region was in different positions in p4568, p5081 and p3778 and occasionally inverted, accompanying an inversion of *wbuC*. In p5081, it positioned at 8–11 kb and remnants of its previous position were seen downstream at 76 kb. In IncI1/*bla*_CTX-M-14_ plasmids, *bla*_CTX-M-14_ was downstream of IS110.

Integrons are additional genetic elements thought to be important in the spread of cefotaximases. Particularly, they are thought to be important in the zoonotic transfer of CTX-M to humans; where ESBL is co-localized on the same plasmid, they may be co-selected in the gastrointestinal tract of livestock which are treated with antibiotics [54]. Class 1 integron *IntI1* was present in both IncI1 *bla*_CTX-M-1_ and IncI1 *bla*_CTX-M-14_ plasmids. It is well documented in Gram-negative micro-organisms and often contains an *aadA* resistance determinant which encodes streptomycin resistance [55]. Four of the IncI1/*bla*_CTX-M-1_ plasmids exhibited this *IntI1-aadA* gene cassette (pPE13-096, p1796, p2388 and p3935). These were either accompanied by *xer_1* or *xer_D*, although all the IncI1/*bla*_CTX-M-1_ plasmids contained genes encoding tyrosine recombinase (variants 1, 2 or D). Class 1 integrons are usually more prevalent in human-influenced environments, such as human-enteric isolates, along with the *sul1* gene, which mediates sulphonamide resistance. The *intI1* containing IncI1 plasmids in this study also encoded *sul2,* which confers sulphonamide resistance but it was located 3.9–5.1 kb apart from the IntI1 integron. *bla*_CTX-M-14_ plasmids, by contrast, all had variants of *xerC* tyrosine recombinase and *aph(3ʺ)-Ib* (which confers resistance to aminoglycosides) and a dihydropteroate synthase, upstream of IS110.

The prevalence of IncI1/*bla*_CTX-M-1_ plasmids belonging to the green clade was very high (894/1,379 isolates), being much higher than the IncI1/*bla*_CTX-M-14_, which indicates that certain features of these plasmids are adaptive for the *E. coli* host, especially those from livestock. Since bacteria typically incur a cost for harbouring plasmids (the energetic cost of conjugation is high), adaptive traits conferred by plasmid carriage must ameliorate these costs. The UK has seen a wide reduction in antibiotic usage in livestock as part of an ongoing antibiotic stewardship programme and this has been accompanied by a reduction in circulating resistance. The data presented here also suggests that the number of circulating cefotaximases on IncI1 plasmids has decreased overall in recent years. Nevertheless, their persistence and stability in different host species in samples collected over a number of years are noticeable and necessitate further monitoring using resolved plasmid databases, such as the one used here to search for plasmids belonging to this group, to inform future monitoring efforts. Such precision genomic monitoring would improve our understanding of persistence of cefotaximases on mobile elements in livestock, even with decreased selection pressure from antimicrobial usage. It would be prudent to complete the same level of analysis on the other ESC and plasmid combinations, such as IncF and IncX that we are recovering more frequently in recent years.

## Supplementary material

10.1099/mgen.0.001775 Fig. S1.

## Abbreviations

AMR: antimicrobial resistance
APHA: Animal and Plant Health Agency
ESBL: extended spectrum beta lactamases
ESCs: extended-spectrum cephalosporinase
HP-CIA: highest priority critically important antimicrobial
IS: insertion sequence
LB: Lysogeny broth
LB-G: lysogeny broth without glucose
MRR: multidrug resistance region
