# Supplementary material for: Stabilizing features of cefotaximase harbouring plasmids enable persistence in UK livestock
Source: Microb Genom. 2026 Jul 7;12(7):001775. doi: 10.1099/mgen.0.001775 (PMC13341076; doi:10.1099/mgen.0.001775)
Supplement: Fig. S1. [file mgen-12-01775-s001.pdf]

Supplementary Materials

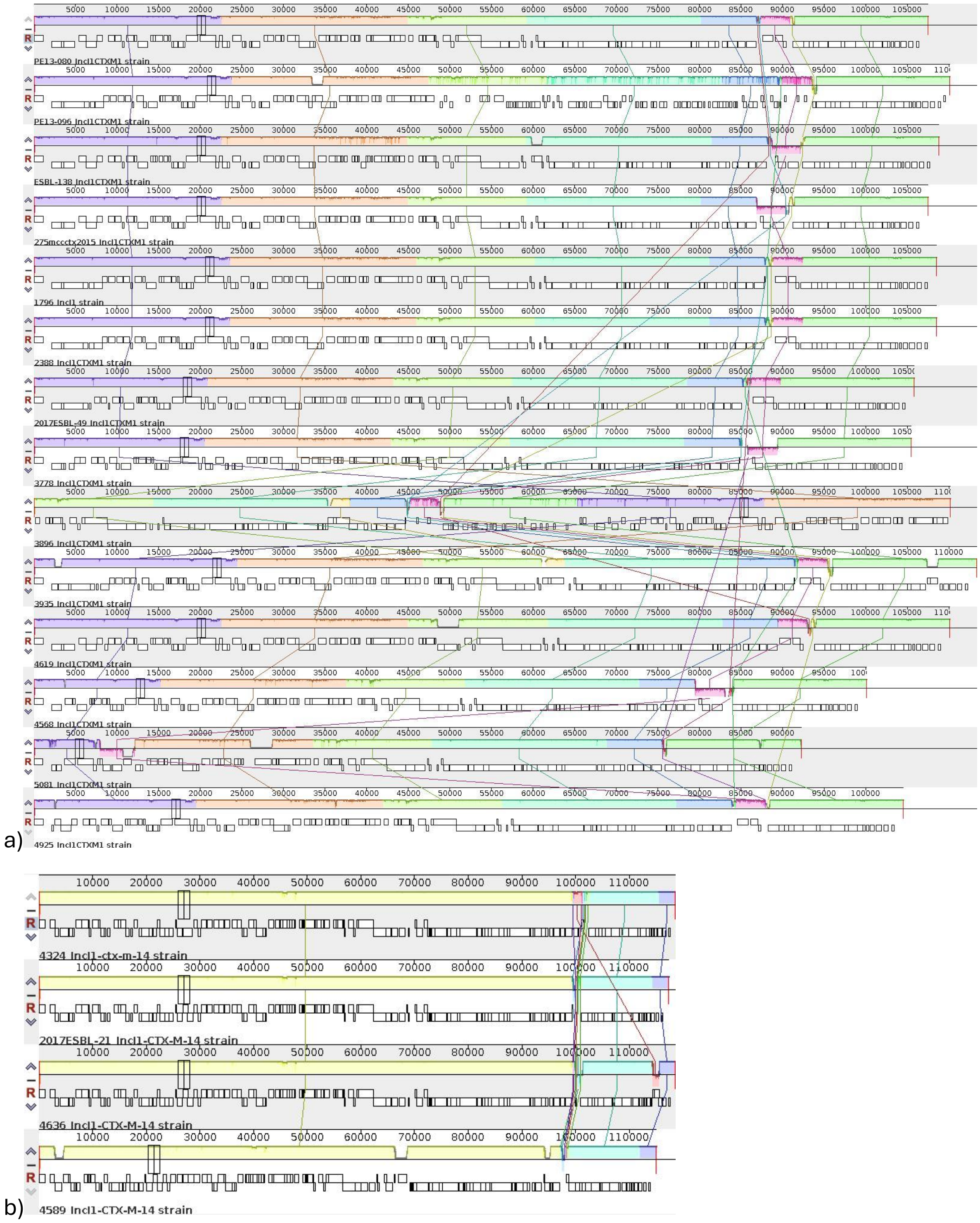

Supplementary Figure 1) a) Mauve progressive alignment of IncI1/CTX-M-1 plasmids and **b)** IncI1/CTX-M-14 plasmids, ordered by year of sample collection. Inversions are denoted where coloured regions are below the centre line (black). Homologous genes are shown by coloured lines.

Supplementary Table 1 i) The number of isolates with each plasmid replicon (by incompatibility group) by animal/meat and **ii)** the number of isolates with each plasmid replicon type per year. Numbers above are actual numbers and below are percentages of total isolates from that animal/meat. IncF plasmids were the most common plasmid, present in 83.9% of isolates, followed by IncI1 (67%), Col (65.92%) and IncX (17.55%). Col plasmids typically do not harbour AMR genes, so this study focused on IncF, IncI1 and IncX.

i)

	Animal								
**Replicon type**	**Beef**	**Broiler**	**Chicken**	**Lamb**	**Pig**	**Pork**	**Turkey-C**	**Turkey-M**	**Total**
IncF	4	227	353	3	486	13	38	33	1157
	80	89.72	86.31	100.00	81.54	92.86	76.00	67.35	83.90
IncI1	2	183	290	0	396	10	27	2	910
	40	72.33	70.90	0.00	66.44	71.43	54.00	4.08	65.99
IncX	0	49	36	0	137	0	9	11	242
	0.00	19.37	8.80	0.00	22.99	0.00	18.00	22.45	17.55
IncAC	0	0	0	0	1	0	0	0	0
	0.00	19.37	8.80	0.00	22.99	0.00	18.00	22.45	17.55
IncR	0	0	0	0	5	0	0	0	2
	0.00	0.40	0.00	0.00	0.84	0.00	0.00	0.00	0.44
IncQ	0	4	7	2	86	0	5	0	104
	0.00	1.98	2.20	0.00	6.04	0.00	2.00	0.00	3.70
IncN	0	5	9	0	36	0	1	0	51
	0.00	1.58	1.71	66.67	14.43	0.00	10.00	0.00	7.54
IncBOKZ	2	63	113	0	22	0	0	0	200
	40.00	24.90	27.63	0.00	3.69	0.00	0.00	0.00	14.50
HI	0	16	23	0	34	0	2	2	77
	0.00	6.32	5.62	0.00	5.70	0.00	4.00	4.08	5.58
Col	4	184	247	2	399	12	36	25	909
	80.00	72.73	60.39	66.67	66.95	85.71	72.00	51.02	65.92
p0111	1	64	80	0	50	0	18	15	228
	20.00	25.30	19.56	0.00	8.39	0.00	36.00	30.61	16.53
IncY	0	5	16	0	107	2	3	0	133
	0.00	1.98	3.91	0.00	17.95	14.29	6.00	0.00	9.64
IncI2	0	5	5	0	1	7	2	8	3
	0.00	1.98	3.91	0.00	17.95	14.29	6.00	0.00	9.64

ii)

	Year							
Replicon type	2013	2015	2016	2017	2018	2019	2020	Total
IncF	144	163	389	109	119	87	146	1157
	77.84	84.02	87.42	83.85	90.84	82.08	77.66	83.90
IncI1	130	132	352	81	96	65	54	910
	70.27	68.04	17.78	62.31	73.28	61.32	28.72	65.99
IncX	46	39	68	33	13	19	24	242
	24.86	20.10	3.43	25.38	9.92	17.92	12.77	17.55
IncAC	1	2	1	0	0	2	0	6
	0.54	1.03	0.05	0.00	0.00	1.89	0.00	0.44
IncR	14	4	0	11	0	2	1	32
	7.57	2.06	0.00	8.46	0.00	1.89	0.53	2.32
IncQ	20	30	9	22	7	14	2	104
	10.81	15.46	0.45	16.92	5.34	13.21	1.06	7.54
IncN	20	9	3	6	0	1	12	51
	10.81	4.64	0.00	4.62	0.00	0.94	6.38	3.70
IncBOKZ	4	3	140	9	27	8	9	200
	2.16	1.55	0.00	6.92	20.61	7.55	4.79	14.50
HI	8	16	31	7	7	3	5	77
	4.32	8.25	6.97	5.38	5.34	2.83	2.66	5.58
Col	123	127	358	94	80	71	56	909
	66.49	65.46	0.00	72.31	61.07	66.98	29.79	65.92
p0111	14	10	98	15	29	12	50	228
	7.57	5.15	0.00	11.54	22.14	11.32	26.60	16.53
IncY	33	30	8	25	7	21	9	133
	17.84	15.46	0.00	19.23	5.34	19.81	4.79	9.64
IncI2	1	1	21	3	11	2	7	46
	0.54	0.52	4.72	2.31	8.40	1.89	3.72	3.34

Supplementary table 2) i) The number of isolates with each cefotaximase (by incompatibility group) by animal/meat. **ii)** The number of contigs containing different beat-lactamases in the data. CTX-M is the most prevalent, followed by TEM, CMY and SHV. **iii)** most common sequence types, their host species and years identified of isolates harbouring IncI1/$bla_{\text{CTX-M-1}}$. **iv)** the number of isolates with each cefotaximase per year. Numbers above are actual numbers and below are percentages of total isolates from that animal/meat. $bla_{\text{CTX-M-1}}$ was the most common cefotaximase, present in 57.14% of all isolates followed by $bla_{\text{CTX-M-15}}$ (7.69%), $bla_{\text{CTX-M-55}}$ (7.25%) and $bla_{\text{CTX-M-14}}$ (2.68%).

i)

	Year							
bla-CTX-M variant	2013	2015	2016	2017	2018	2019	2020	Total
bla-CTX-M-1	141	113	302	68	75	59	30	788
	76.22	58.25	67.87	52.31	57.25	55.66	15.96	57.14
bla-CTX-M-15	16	20	6	12	1	17	34	106
	8.65	10.31	1.35	9.23	0.76	16.04	18.09	7.69
bla-CTX-M-55	4	10	9	5	1	3	68	100
	2.16	5.15	2.02	3.85	0.76	2.83	36.17	7.25
bla-CTX-M-14	3	8	2	8	5	10	1	37
	1.62	4.12	0.45	6.15	3.82	9.43	0.53	2.68

ii)

	beta-lactamase group							
	CTXM	CMY	OXA	DHA	LAP-2	SHV	TEM	ampC
**No.Isolates**	1076	155	21	5	15	106	384	44

iii)

MLST	Total isolates	Host species	Years
602	51	Beef, Broiler, Chicken, Pig, Pork, Turkey-C	2013-2020
57	20	Broiler, Chicken, Pig	2013-2018
101	17	Broiler, Chicken, Pig, Pork	2013-2019
10	16	Broiler, Chicken, Pig, Turkey-C	2013-2018
117	16	Broiler, Chicken, Pig	2013-2017
350	15	Broiler, Chicken	2016-2018
38	12	Broiler, Chicken	2016-2018
23	11	Chicken, Pig, Turkey-M	2013-2020
410	9	Pig, Pork, Turkey-C	2013-2019
58	5	Chicken, Pig	2013-2019

iv)

	Animal								
bla-CTX-M variant	Beef	Broiler	Chicken	Lamb	Pig	Pork	Turkey-C	Turkey-M	Total
bla-CTX-M-1	2	140	242	0	370	9	24	1	788
	40.00	55.34	59.17	0.00	62.08	64.29	48.00	2.04	57.14
bla-CTX-M-15	0	2	4	2	65	0	4	29	106
	0.00	0.79	0.98	66.67	10.91	0.00	8.00	59.18	7.69
bla-CTX-M-55	0	18	47	0	22	0	1	12	100
	0.00	7.11	11.49	0.00	3.69	0.00	2.00	24.49	7.25
bla-CTX-M-14	1	2	1	0	26	2	5	0	37
	20.00	0.79	0.24	0.00	4.36	14.29	10.00	0.00	2.68

Finding plasmids using BLASTn – Validation.

BLASTn was used to find the top hits for the circularised IncI1 $_{blaCTX-M-1/blaCTX-M-14}$ plasmids in the short-read assemblies identified as positive for bearing IncI1 plasmids (n=924) with an identity >=99%, a cumulative coverage of >=98% and a minimum length of 1000bp. This method was validated in two ways: i) it was verified that BLASTn at these thresholds could correctly identify plasmids in 'self' isolates and ii) alignment was conducted to verify that the contig hits for the top plasmids were covering large portions of the plasmid (Supplementary figure 3). Out of the panel of 18 plasmids, this method correctly identified the plasmid in the 'self' strain 15 times as the top hit and twice as the second-top hit. p3935 was not correctly identified in the 'self' strain, however upon further investigation, the short – read assembly quality for this strain was poor, relative to the other isolates.

Supplementary table 3 – list of study and corresponding sample accessions used for the analysis

Study accession	Sample accession
PRJEB34493	ERR3531128
PRJEB34493	ERR3531129
PRJEB34493	ERR3531130
PRJEB34493	ERR3531138
PRJEB34493	ERR3531144
PRJEB34493	ERR3531147
PRJEB34493	ERR3531150
PRJEB34493	ERR3531153
PRJEB34493	ERR3531157
PRJEB34493	ERR3531160
PRJEB34493	ERR3531167
PRJEB34493	ERR3531169
PRJEB34493	ERR3531170
PRJEB34493	ERR3531171
PRJEB34493	ERR3531172
PRJEB34493	ERR3531176
PRJEB34493	ERR3531177
PRJEB34493	ERR3531178
PRJEB34493	ERR3531181
PRJEB34493	ERR3531182
PRJEB34493	ERR3531183
PRJEB34493	ERR3531184
PRJEB34493	ERR3531191
PRJEB34493	ERR3531196
PRJEB34493	ERR3531200
PRJEB34493	ERR3531201
PRJEB34493	ERR3531205
PRJEB34493	ERR3531211
PRJEB34493	ERR3531213
PRJEB34493	ERR3531216
PRJEB34493	ERR3531219
PRJEB34493	ERR3531223
PRJEB34493	ERR3531224
PRJEB34493	ERR3531227
PRJEB34493	ERR3531231
PRJEB34493	ERR3531238
PRJEB34493	ERR3531240
PRJEB34493	ERR3531241
PRJEB34493	ERR3531244
PRJEB34493	ERR3531251
PRJEB34493	ERR3531258
PRJEB34493	ERR3531260
PRJEB34493	ERR3531261
PRJEB34493	ERR3531262
PRJEB34493	ERR3531264
PRJEB34493	ERR3531266
PRJEB34493	ERR3531269
PRJEB34493	ERR3531274
PRJEB34493	ERR3531275

PRJEB34493	ERR3531278
PRJEB34493	ERR3531295
PRJEB34493	ERR3531296
PRJEB34493	ERR3531297
PRJEB34493	ERR3531300
PRJEB34493	ERR3531304
PRJEB34493	ERR3531305
PRJEB34493	ERR3531307
PRJEB34493	ERR3531314
PRJEB34493	ERR3531328
PRJEB34493	ERR3531335
PRJEB34493	ERR3531338
PRJEB34493	ERR3531339
PRJEB34493	ERR3531341
PRJEB34493	ERR3531342
PRJEB34493	ERR3531344
PRJEB34493	ERR3531345
PRJEB34493	ERR3531346
PRJEB34493	ERR3531348
PRJEB34493	ERR3531349
PRJEB34493	ERR3531363
PRJEB34493	ERR3531364
PRJEB34493	ERR3531365
PRJEB34493	ERR3531367
PRJEB34493	ERR3531368
PRJEB34493	ERR3531373
PRJEB34493	ERR3531380
PRJEB34493	ERR3531381
PRJEB34493	ERR3531383
PRJEB34493	ERR3531384
PRJEB34493	ERR3531385
PRJEB34493	ERR3531386
PRJEB34493	ERR3531389
PRJEB34493	ERR3531391
PRJEB34493	ERR3531393
PRJEB34493	ERR3531395
PRJEB34493	ERR3531397
PRJEB34493	ERR3531399
PRJEB34493	ERR3531402
PRJEB34493	ERR3531406
PRJEB34493	ERR3531408
PRJEB34493	ERR3531418
PRJEB34493	ERR3531424
PRJEB34493	ERR3531430
PRJEB34493	ERR3531431
PRJEB34493	ERR3531436
PRJEB34493	ERR3531441
PRJEB34493	ERR3531442
PRJEB34493	ERR3531450
PRJEB34493	ERR3531451
PRJEB34493	ERR3531452

PRJEB34493	ERR3531454
PRJEB34493	ERR3531455
PRJEB34493	ERR3531456
PRJEB34493	ERR3531458
PRJEB34493	ERR3531461
PRJEB34493	ERR3531465
PRJEB34493	ERR3531466
PRJEB34493	ERR3531468
PRJEB34493	ERR3531470
PRJEB34493	ERR3531473
PRJEB34493	ERR3531475
PRJEB34493	ERR3531476
PRJEB34493	ERR3531478
PRJEB34493	ERR3531482
PRJEB34493	ERR3531484
PRJEB34493	ERR3531486
PRJEB34493	ERR3531495
PRJEB34493	ERR3531497
PRJEB34493	ERR3531499
PRJEB34493	ERR3531501
PRJEB34493	ERR3531502
PRJEB34493	ERR3531508
PRJEB34493	ERR3531509
PRJEB34493	ERR3531512
PRJEB34493	ERR3531517
PRJEB34493	ERR3531520
PRJEB34493	ERR3531523
PRJEB34493	ERR3531527
PRJEB34493	ERR3531531
PRJEB34493	ERR3531533
PRJEB34493	ERR3531538
PRJEB34493	ERR3531543
PRJEB34493	ERR3531545
PRJEB34493	ERR3531548
PRJEB34493	ERR3531556
PRJEB34493	ERR3531561
PRJEB34493	ERR3531563
PRJEB34493	ERR3531565
PRJEB34493	ERR3531567
PRJEB34493	ERR3531568
PRJEB34493	ERR3531575
PRJEB34493	ERR3531579
PRJEB34493	ERR3531580
PRJEB34493	ERR3531581
PRJEB34493	ERR3531583
PRJEB34493	ERR3531585
PRJEB34493	ERR3531587
PRJEB34493	ERR3531594
PRJEB34493	ERR3531596
PRJEB34493	ERR3531600
PRJEB34493	ERR3531601

PRJEB34493	ERR3531602
PRJEB34493	ERR3531604
PRJEB34493	ERR3531607
PRJEB34493	ERR3531610
PRJEB34493	ERR3531611
PRJEB34493	ERR3531614
PRJEB34493	ERR3531615
PRJEB34493	ERR3531617
PRJEB34493	ERR3531619
PRJEB34493	ERR3531622
PRJEB34493	ERR3531623
PRJEB34493	ERR3531624
PRJEB34493	ERR4656904
PRJEB34493	ERR3531132
PRJEB34493	ERR3531133
PRJEB34493	ERR3531134
PRJEB34493	ERR3531135
PRJEB34493	ERR3531136
PRJEB34493	ERR3531139
PRJEB34493	ERR3531141
PRJEB34493	ERR3531142
PRJEB34493	ERR3531145
PRJEB34493	ERR3531146
PRJEB34493	ERR3531148
PRJEB34493	ERR3531155
PRJEB34493	ERR3531158
PRJEB34493	ERR3531161
PRJEB34493	ERR3531165
PRJEB34493	ERR3531166
PRJEB34493	ERR3531168
PRJEB34493	ERR3531173
PRJEB34493	ERR3531175
PRJEB34493	ERR3531179
PRJEB34493	ERR3531180
PRJEB34493	ERR3531185
PRJEB34493	ERR3531189
PRJEB34493	ERR3531193
PRJEB34493	ERR3531195
PRJEB34493	ERR3531197
PRJEB34493	ERR3531202
PRJEB34493	ERR3531203
PRJEB34493	ERR3531204
PRJEB34493	ERR3531207
PRJEB34493	ERR3531208
PRJEB34493	ERR3531228
PRJEB34493	ERR3531229
PRJEB34493	ERR3531233
PRJEB34493	ERR3531234
PRJEB34493	ERR3531235
PRJEB34493	ERR3531242
PRJEB34493	ERR3531245

PRJEB34493	ERR3531246
PRJEB34493	ERR3531247
PRJEB34493	ERR3531248
PRJEB34493	ERR3531249
PRJEB34493	ERR3531253
PRJEB34493	ERR3531255
PRJEB34493	ERR3531259
PRJEB34493	ERR3531263
PRJEB34493	ERR3531265
PRJEB34493	ERR3531267
PRJEB34493	ERR3531276
PRJEB34493	ERR3531277
PRJEB34493	ERR3531279
PRJEB34493	ERR3531280
PRJEB34493	ERR3531281
PRJEB34493	ERR3531285
PRJEB34493	ERR3531290
PRJEB34493	ERR3531292
PRJEB34493	ERR3531294
PRJEB34493	ERR3531298
PRJEB34493	ERR3531299
PRJEB34493	ERR3531301
PRJEB34493	ERR3531306
PRJEB34493	ERR3531308
PRJEB34493	ERR3531311
PRJEB34493	ERR3531312
PRJEB34493	ERR3531313
PRJEB34493	ERR3531317
PRJEB34493	ERR3531318
PRJEB34493	ERR3531319
PRJEB34493	ERR3531320
PRJEB34493	ERR3531321
PRJEB34493	ERR3531322
PRJEB34493	ERR3531330
PRJEB34493	ERR3531331
PRJEB34493	ERR3531332
PRJEB34493	ERR3531333
PRJEB34493	ERR3531337
PRJEB34493	ERR3531343
PRJEB34493	ERR3531347
PRJEB34493	ERR3531351
PRJEB34493	ERR3531355
PRJEB34493	ERR3531356
PRJEB34493	ERR3531357
PRJEB34493	ERR3531359
PRJEB34493	ERR3531362
PRJEB34493	ERR3531366
PRJEB34493	ERR3531369
PRJEB34493	ERR3531371
PRJEB34493	ERR3531372
PRJEB34493	ERR3531374

PRJEB34493	ERR3531375
PRJEB34493	ERR3531378
PRJEB34493	ERR3531382
PRJEB34493	ERR3531392
PRJEB34493	ERR3531400
PRJEB34493	ERR3531403
PRJEB34493	ERR3531404
PRJEB34493	ERR3531407
PRJEB34493	ERR3531409
PRJEB34493	ERR3531414
PRJEB34493	ERR3531416
PRJEB34493	ERR3531422
PRJEB34493	ERR3531426
PRJEB34493	ERR3531428
PRJEB34493	ERR3531429
PRJEB34493	ERR3531433
PRJEB34493	ERR3531434
PRJEB34493	ERR3531437
PRJEB34493	ERR3531444
PRJEB34493	ERR3531448
PRJEB34493	ERR3531453
PRJEB34493	ERR3531459
PRJEB34493	ERR3531463
PRJEB34493	ERR3531467
PRJEB34493	ERR3531469
PRJEB34493	ERR3531474
PRJEB34493	ERR3531479
PRJEB34493	ERR3531480
PRJEB34493	ERR3531488
PRJEB34493	ERR3531491
PRJEB34493	ERR3531492
PRJEB34493	ERR3531496
PRJEB34493	ERR3531498
PRJEB34493	ERR3531503
PRJEB34493	ERR3531504
PRJEB34493	ERR3531505
PRJEB34493	ERR3531506
PRJEB34493	ERR3531507
PRJEB34493	ERR3531510
PRJEB34493	ERR3531511
PRJEB34493	ERR3531519
PRJEB34493	ERR3531525
PRJEB34493	ERR3531528
PRJEB34493	ERR3531529
PRJEB34493	ERR3531532
PRJEB34493	ERR3531534
PRJEB34493	ERR3531535
PRJEB34493	ERR3531536
PRJEB34493	ERR3531537
PRJEB34493	ERR3531539
PRJEB34493	ERR3531546

PRJEB34493	ERR3531549
PRJEB34493	ERR3531554
PRJEB34493	ERR3531557
PRJEB34493	ERR3531562
PRJEB34493	ERR3531566
PRJEB34493	ERR3531574
PRJEB34493	ERR3531576
PRJEB34493	ERR3531577
PRJEB34493	ERR3531578
PRJEB34493	ERR3531127
PRJEB34493	ERR3531588
PRJEB34493	ERR3531590
PRJEB34493	ERR3531131
PRJEB34493	ERR3531591
PRJEB34493	ERR3531137
PRJEB34493	ERR3531140
PRJEB34493	ERR3531592
PRJEB34493	ERR3531593
PRJEB34493	ERR3531143
PRJEB34493	ERR3531149
PRJEB34493	ERR3531595
PRJEB34493	ERR3531151
PRJEB34493	ERR3531598
PRJEB34493	ERR3531152
PRJEB34493	ERR3531609
PRJEB34493	ERR3531612
PRJEB34493	ERR3531154
PRJEB34493	ERR3531613
PRJEB34493	ERR3531156
PRJEB34493	ERR3531159
PRJEB34493	ERR3531618
PRJEB34493	ERR3531621
PRJEB34493	ERR3531162
PRJEB34493	ERR4656838
PRJEB34493	ERR3531163
PRJEB34493	ERR4656899
PRJEB34493	ERR3531164
PRJEB34493	ERR4656924
PRJEB34493	ERR3531174
PRJEB34493	ERR3531186
PRJEB34493	ERR3531187
PRJEB34493	ERR3531188
PRJEB34493	ERR3531190
PRJEB34493	ERR3531192
PRJEB34493	ERR3531194
PRJEB34493	ERR3531198
PRJEB34493	ERR3531199
PRJEB34493	ERR3531206
PRJEB34493	ERR3531209
PRJEB34493	ERR3531210
PRJEB34493	ERR3531212

PRJEB34493	ERR3531214
PRJEB34493	ERR3531215
PRJEB34493	ERR3531217
PRJEB34493	ERR3531218
PRJEB34493	ERR3531220
PRJEB34493	ERR3531221
PRJEB34493	ERR3531222
PRJEB34493	ERR3531225
PRJEB34493	ERR3531226
PRJEB34493	ERR3531230
PRJEB34493	ERR3531232
PRJEB34493	ERR3531236
PRJEB34493	ERR3531237
PRJEB34493	ERR3531239
PRJEB34493	ERR3531243
PRJEB34493	ERR3531250
PRJEB34493	ERR3531252
PRJEB34493	ERR3531254
PRJEB34493	ERR3531256
PRJEB34493	ERR3531257
PRJEB34493	ERR3531268
PRJEB34493	ERR3531270
PRJEB34493	ERR3531271
PRJEB34493	ERR3531272
PRJEB34493	ERR3531273
PRJEB34493	ERR3531282
PRJEB34493	ERR3531283
PRJEB34493	ERR3531284
PRJEB34493	ERR3531286
PRJEB34493	ERR3531287
PRJEB34493	ERR3531288
PRJEB34493	ERR3531289
PRJEB34493	ERR3531291
PRJEB34493	ERR3531293
PRJEB34493	ERR3531302
PRJEB34493	ERR3531303
PRJEB34493	ERR3531309
PRJEB34493	ERR3531310
PRJEB34493	ERR3531315
PRJEB34493	ERR3531316
PRJEB34493	ERR3531323
PRJEB34493	ERR3531324
PRJEB34493	ERR3531325
PRJEB34493	ERR3531326
PRJEB34493	ERR3531327
PRJEB34493	ERR3531329
PRJEB34493	ERR3531334
PRJEB34493	ERR3531336
PRJEB34493	ERR3531340
PRJEB34493	ERR3531350
PRJEB34493	ERR3531352

PRJEB34493	ERR3531353
PRJEB34493	ERR3531354
PRJEB34493	ERR3531358
PRJEB34493	ERR3531360
PRJEB34493	ERR3531361
PRJEB34493	ERR3531370
PRJEB34493	ERR3531376
PRJEB34493	ERR3531377
PRJEB34493	ERR3531379
PRJEB34493	ERR3531387
PRJEB34493	ERR3531388
PRJEB34493	ERR3531390
PRJEB34493	ERR3531394
PRJEB34493	ERR3531396
PRJEB34493	ERR3531398
PRJEB34493	ERR3531401
PRJEB34493	ERR3531405
PRJEB34493	ERR3531410
PRJEB34493	ERR3531411
PRJEB34493	ERR3531412
PRJEB34493	ERR3531413
PRJEB34493	ERR3531415
PRJEB34493	ERR3531417
PRJEB34493	ERR3531419
PRJEB34493	ERR3531420
PRJEB34493	ERR3531421
PRJEB34493	ERR3531423
PRJEB34493	ERR3531425
PRJEB34493	ERR3531427
PRJEB34493	ERR3531432
PRJEB34493	ERR3531435
PRJEB34493	ERR3531438
PRJEB34493	ERR3531439
PRJEB34493	ERR3531440
PRJEB34493	ERR3531443
PRJEB34493	ERR3531445
PRJEB34493	ERR3531446
PRJEB34493	ERR3531447
PRJEB34493	ERR3531449
PRJEB34493	ERR3531457
PRJEB34493	ERR3531460
PRJEB34493	ERR3531462
PRJEB34493	ERR3531464
PRJEB34493	ERR3531471
PRJEB34493	ERR3531472
PRJEB34493	ERR3531477
PRJEB34493	ERR3531481
PRJEB34493	ERR3531483
PRJEB34493	ERR3531485
PRJEB34493	ERR3531487
PRJEB34493	ERR3531489

PRJEB34493	ERR3531490
PRJEB34493	ERR3531493
PRJEB34493	ERR3531494
PRJEB34493	ERR3531500
PRJEB34493	ERR3531513
PRJEB34493	ERR3531514
PRJEB34493	ERR3531515
PRJEB34493	ERR3531516
PRJEB34493	ERR3531518
PRJEB34493	ERR3531521
PRJEB34493	ERR3531522
PRJEB34493	ERR3531524
PRJEB34493	ERR3531526
PRJEB34493	ERR3531530
PRJEB34493	ERR3531540
PRJEB34493	ERR3531541
PRJEB34493	ERR3531542
PRJEB34493	ERR3531544
PRJEB34493	ERR3531547
PRJEB34493	ERR3531550
PRJEB34493	ERR3531551
PRJEB34493	ERR3531552
PRJEB34493	ERR3531553
PRJEB34493	ERR3531555
PRJEB34493	ERR3531558
PRJEB34493	ERR3531559
PRJEB34493	ERR3531560
PRJEB34493	ERR3531564
PRJEB34493	ERR3531569
PRJEB34493	ERR3531570
PRJEB34493	ERR3531571
PRJEB34493	ERR3531572
PRJEB34493	ERR3531573
PRJEB34493	ERR3531582
PRJEB34493	ERR3531584
PRJEB34493	ERR3531586
PRJEB34493	ERR3531589
PRJEB34493	ERR3531597
PRJEB34493	ERR3531599
PRJEB34493	ERR3531603
PRJEB34493	ERR3531605
PRJEB34493	ERR3531606
PRJEB34493	ERR3531608
PRJEB34493	ERR3531616
PRJEB34493	ERR3531620
PRJEB34493	ERR4656897
PRJEB34493	ERR4656923
PRJEB67810	ERR12422589
PRJEB67810	ERR12422590
PRJEB67810	ERR12422593
PRJEB67810	ERR12422594

PRJEB67810	ERR12422595
PRJEB67810	ERR12422597
PRJEB67810	ERR12422599
PRJEB67810	ERR12422604
PRJEB67810	ERR12422607
PRJEB67810	ERR12422608
PRJEB67810	ERR12422612
PRJEB67810	ERR12422616
PRJEB67810	ERR12422617
PRJEB67810	ERR12422625
PRJEB67810	ERR12422630
PRJEB67810	ERR12422631
PRJEB67810	ERR12422633
PRJEB67810	ERR12422636
PRJEB67810	ERR12422638
PRJEB67810	ERR12422641
PRJEB67810	ERR12422644
PRJEB67810	ERR12422648
PRJEB67810	ERR12422651
PRJEB67810	ERR12422653
PRJEB67810	ERR12422656
PRJEB67810	ERR12422657
PRJEB67810	ERR12422658
PRJEB67810	ERR12422673
PRJEB67810	ERR12422675
PRJEB67810	ERR12422677
PRJEB67810	ERR12422685
PRJEB67810	ERR12422689
PRJEB67810	ERR12422691
PRJEB67810	ERR12422703
PRJEB67810	ERR12422707
PRJEB67810	ERR12422715
PRJEB67810	ERR12422722
PRJEB67810	ERR12422725
PRJEB67810	ERR12422727
PRJEB67810	ERR12422732
PRJEB67810	ERR12422737
PRJEB67810	ERR12422738
PRJEB67810	ERR12422744
PRJEB67810	ERR12422748
PRJEB67810	ERR12422750
PRJEB67810	ERR12422753
PRJEB67810	ERR12422754
PRJEB67810	ERR12422761
PRJEB67810	ERR12422762
PRJEB67810	ERR12422766
PRJEB67810	ERR12422779
PRJEB67810	ERR12422784
PRJEB67810	ERR12422787
PRJEB67810	ERR12422788
PRJEB67810	ERR12422802

PRJEB67810	ERR12422807
PRJEB67810	ERR12422813
PRJEB67810	ERR12422820
PRJEB67810	ERR12422821
PRJEB67810	ERR12422829
PRJEB67810	ERR12422832
PRJEB67810	ERR12422833
PRJEB67810	ERR12422836
PRJEB67810	ERR12422844
PRJEB67810	ERR12422846
PRJEB67810	ERR12422849
PRJEB67810	ERR12422852
PRJEB67810	ERR12422853
PRJEB67810	ERR12422855
PRJEB67810	ERR12422858
PRJEB67810	ERR12422860
PRJEB67810	ERR12422862
PRJEB67810	ERR12422864
PRJEB67810	ERR12422868
PRJEB67810	ERR12422871
PRJEB67810	ERR12422872
PRJEB67810	ERR12422876
PRJEB67810	ERR12422878
PRJEB67810	ERR12422879
PRJEB67810	ERR12422885
PRJEB67810	ERR12422886
PRJEB67810	ERR12422888
PRJEB67810	ERR12422890
PRJEB67810	ERR12422896
PRJEB67810	ERR12422900
PRJEB67810	ERR12422901
PRJEB67810	ERR12422902
PRJEB67810	ERR12422909
PRJEB67810	ERR12422916
PRJEB67810	ERR12422918
PRJEB67810	ERR12422919
PRJEB67810	ERR12422920
PRJEB67810	ERR12422921
PRJEB67810	ERR12422923
PRJEB67810	ERR12422925
PRJEB67810	ERR12422926
PRJEB67810	ERR12422927
PRJEB67810	ERR12422929
PRJEB67810	ERR12422932
PRJEB67810	ERR12422942
PRJEB67810	ERR12422945
PRJEB67810	ERR12422946
PRJEB67810	ERR12422949
PRJEB67810	ERR12422951
PRJEB67810	ERR12422956
PRJEB67810	ERR12422959

PRJEB67810	ERR12422963
PRJEB67810	ERR12422964
PRJEB67810	ERR12422966
PRJEB67810	ERR12422967
PRJEB67810	ERR12422971
PRJEB67810	ERR12422973
PRJEB67810	ERR12422978
PRJEB67810	ERR12422979
PRJEB67810	ERR12422985
PRJEB67810	ERR12422987
PRJEB67810	ERR12422988
PRJEB67810	ERR12422990
PRJEB67810	ERR12422991
PRJEB67810	ERR12422994
PRJEB67810	ERR12423000
PRJEB67810	ERR12423005
PRJEB67810	ERR12423007
PRJEB67810	ERR12423008
PRJEB67810	ERR12423010
PRJEB67810	ERR12423012
PRJEB67810	ERR12423015
PRJEB67810	ERR12423019
PRJEB67810	ERR12423020
PRJEB67810	ERR12423027
PRJEB67810	ERR12423037
PRJEB67810	ERR12423038
PRJEB67810	ERR12423046
PRJEB67810	ERR12423050
PRJEB67810	ERR12423051
PRJEB67810	ERR12423056
PRJEB67810	ERR12423060
PRJEB67810	ERR12423061
PRJEB67810	ERR12423064
PRJEB67810	ERR12423067
PRJEB67810	ERR12423071
PRJEB67810	ERR12423073
PRJEB67810	ERR12423074
PRJEB67810	ERR12423076
PRJEB67810	ERR12423078
PRJEB67810	ERR12423083
PRJEB67810	ERR12423086
PRJEB67810	ERR12423088
PRJEB67810	ERR12423089
PRJEB67810	ERR12423091
PRJEB67810	ERR12423097
PRJEB67810	ERR12423098
PRJEB67810	ERR12423100
PRJEB67810	ERR12423102
PRJEB67810	ERR12423109
PRJEB67810	ERR12423110
PRJEB67810	ERR12423113

PRJEB67810	ERR12423118
PRJEB67810	ERR12423120
PRJEB67810	ERR12423129
PRJEB67810	ERR12423130
PRJEB67810	ERR12423134
PRJEB67810	ERR12423138
PRJEB67810	ERR12423139
PRJEB67810	ERR12423141
PRJEB67810	ERR12423143
PRJEB67810	ERR12423144
PRJEB67810	ERR12423145
PRJEB67810	ERR12422592
PRJEB67810	ERR12423146
PRJEB67810	ERR12423148
PRJEB67810	ERR12422598
PRJEB67810	ERR12422600
PRJEB67810	ERR12423152
PRJEB67810	ERR12422601
PRJEB67810	ERR12422602
PRJEB67810	ERR12423154
PRJEB67810	ERR12423158
PRJEB67810	ERR12423161
PRJEB67810	ERR12422605
PRJEB67810	ERR12422609
PRJEB67810	ERR12423162
PRJEB67810	ERR12422614
PRJEB67810	ERR12423165
PRJEB67810	ERR12422615
PRJEB67810	ERR12423170
PRJEB67810	ERR12422618
PRJEB67810	ERR12422619
PRJEB67810	ERR12423171
PRJEB67810	ERR12422620
PRJEB67810	ERR12423172
PRJEB67810	ERR12422621
PRJEB67810	ERR12423173
PRJEB67810	ERR12422622
PRJEB67810	ERR12423178
PRJEB67810	ERR12422627
PRJEB67810	ERR12422628
PRJEB67810	ERR12423179
PRJEB67810	ERR12422629
PRJEB67810	ERR12422632
PRJEB67810	ERR12423185
PRJEB67810	ERR12423186
PRJEB67810	ERR12422635
PRJEB67810	ERR12423192
PRJEB67810	ERR12422637
PRJEB67810	ERR12422639
PRJEB67810	ERR12423193
PRJEB67810	ERR12422642

PRJEB67810	ERR12423203
PRJEB67810	ERR12422645
PRJEB67810	ERR12423205
PRJEB67810	ERR12423207
PRJEB67810	ERR12422646
PRJEB67810	ERR12423210
PRJEB67810	ERR12423214
PRJEB67810	ERR12422647
PRJEB67810	ERR12422650
PRJEB67810	ERR12423216
PRJEB67810	ERR12423217
PRJEB67810	ERR12422652
PRJEB67810	ERR12422655
PRJEB67810	ERR12423223
PRJEB67810	ERR12422661
PRJEB67810	ERR12422666
PRJEB67810	ERR12423225
PRJEB67810	ERR12422667
PRJEB67810	ERR12423228
PRJEB67810	ERR12423229
PRJEB67810	ERR12422671
PRJEB67810	ERR12423231
PRJEB67810	ERR12423235
PRJEB67810	ERR12423236
PRJEB67810	ERR12422672
PRJEB67810	ERR12422674
PRJEB67810	ERR12422678
PRJEB67810	ERR12423242
PRJEB67810	ERR12423243
PRJEB67810	ERR12423244
PRJEB67810	ERR12422684
PRJEB67810	ERR12423248
PRJEB67810	ERR12422687
PRJEB67810	ERR12422688
PRJEB67810	ERR12423253
PRJEB67810	ERR12423255
PRJEB67810	ERR12422692
PRJEB67810	ERR12423256
PRJEB67810	ERR12423258
PRJEB67810	ERR12422694
PRJEB67810	ERR12422697
PRJEB67810	ERR12423261
PRJEB67810	ERR12423262
PRJEB67810	ERR12422699
PRJEB67810	ERR12422700
PRJEB67810	ERR12423263
PRJEB67810	ERR12422704
PRJEB67810	ERR12423265
PRJEB67810	ERR12423268
PRJEB67810	ERR12423272
PRJEB67810	ERR12422705

PRJEB67810	ERR12422706
PRJEB67810	ERR12423273
PRJEB67810	ERR12422708
PRJEB67810	ERR12423276
PRJEB67810	ERR12422709
PRJEB67810	ERR12423277
PRJEB67810	ERR12422711
PRJEB67810	ERR12423282
PRJEB67810	ERR12422712
PRJEB67810	ERR12423286
PRJEB67810	ERR12423288
PRJEB67810	ERR12423297
PRJEB67810	ERR12422713
PRJEB67810	ERR12423302
PRJEB67810	ERR12422716
PRJEB67810	ERR12423305
PRJEB67810	ERR12422719
PRJEB67810	ERR12423308
PRJEB67810	ERR12422723
PRJEB67810	ERR12423312
PRJEB67810	ERR12422724
PRJEB67810	ERR12423317
PRJEB67810	ERR12422728
PRJEB67810	ERR12423328
PRJEB67810	ERR12422730
PRJEB67810	ERR12423330
PRJEB67810	ERR12422731
PRJEB67810	ERR12423332
PRJEB67810	ERR12423334
PRJEB67810	ERR12423337
PRJEB67810	ERR12423339
PRJEB67810	ERR12422733
PRJEB67810	ERR12422734
PRJEB67810	ERR12423343
PRJEB67810	ERR12423344
PRJEB67810	ERR12423348
PRJEB67810	ERR12422735
PRJEB67810	ERR12422736
PRJEB67810	ERR12422741
PRJEB67810	ERR12422743
PRJEB67810	ERR12422745
PRJEB67810	ERR12422747
PRJEB67810	ERR12422749
PRJEB67810	ERR12422751
PRJEB67810	ERR12422752
PRJEB67810	ERR12422755
PRJEB67810	ERR12422757
PRJEB67810	ERR12422760
PRJEB67810	ERR12422763
PRJEB67810	ERR12422764
PRJEB67810	ERR12422765

PRJEB67810	ERR12422767
PRJEB67810	ERR12422768
PRJEB67810	ERR12422769
PRJEB67810	ERR12422770
PRJEB67810	ERR12422773
PRJEB67810	ERR12422775
PRJEB67810	ERR12422776
PRJEB67810	ERR12422777
PRJEB67810	ERR12422778
PRJEB67810	ERR12422782
PRJEB67810	ERR12422783
PRJEB67810	ERR12422785
PRJEB67810	ERR12422591
PRJEB67810	ERR12422596
PRJEB67810	ERR12422786
PRJEB67810	ERR12422603
PRJEB67810	ERR12422789
PRJEB67810	ERR12422606
PRJEB67810	ERR12422791
PRJEB67810	ERR12422610
PRJEB67810	ERR12422611
PRJEB67810	ERR12422795
PRJEB67810	ERR12422613
PRJEB67810	ERR12422796
PRJEB67810	ERR12422799
PRJEB67810	ERR12422623
PRJEB67810	ERR12422803
PRJEB67810	ERR12422624
PRJEB67810	ERR12422804
PRJEB67810	ERR12422626
PRJEB67810	ERR12422634
PRJEB67810	ERR12422806
PRJEB67810	ERR12422640
PRJEB67810	ERR12422810
PRJEB67810	ERR12422811
PRJEB67810	ERR12422814
PRJEB67810	ERR12422643
PRJEB67810	ERR12422649
PRJEB67810	ERR12422816
PRJEB67810	ERR12422818
PRJEB67810	ERR12422654
PRJEB67810	ERR12422819
PRJEB67810	ERR12422823
PRJEB67810	ERR12422659
PRJEB67810	ERR12422660
PRJEB67810	ERR12422662
PRJEB67810	ERR12422824
PRJEB67810	ERR12422663
PRJEB67810	ERR12422664
PRJEB67810	ERR12422665
PRJEB67810	ERR12422826

PRJEB67810	ERR12422828
PRJEB67810	ERR12422830
PRJEB67810	ERR12422831
PRJEB67810	ERR12422668
PRJEB67810	ERR12422834
PRJEB67810	ERR12422669
PRJEB67810	ERR12422670
PRJEB67810	ERR12422835
PRJEB67810	ERR12422838
PRJEB67810	ERR12422676
PRJEB67810	ERR12422839
PRJEB67810	ERR12422841
PRJEB67810	ERR12422842
PRJEB67810	ERR12422679
PRJEB67810	ERR12422845
PRJEB67810	ERR12422680
PRJEB67810	ERR12422847
PRJEB67810	ERR12422848
PRJEB67810	ERR12422681
PRJEB67810	ERR12422851
PRJEB67810	ERR12422682
PRJEB67810	ERR12422854
PRJEB67810	ERR12422683
PRJEB67810	ERR12422857
PRJEB67810	ERR12422861
PRJEB67810	ERR12422686
PRJEB67810	ERR12422690
PRJEB67810	ERR12422863
PRJEB67810	ERR12422693
PRJEB67810	ERR12422867
PRJEB67810	ERR12422874
PRJEB67810	ERR12422695
PRJEB67810	ERR12422696
PRJEB67810	ERR12422880
PRJEB67810	ERR12422698
PRJEB67810	ERR12422882
PRJEB67810	ERR12422701
PRJEB67810	ERR12422702
PRJEB67810	ERR12422883
PRJEB67810	ERR12422892
PRJEB67810	ERR12422710
PRJEB67810	ERR12422714
PRJEB67810	ERR12422717
PRJEB67810	ERR12422895
PRJEB67810	ERR12422718
PRJEB67810	ERR12422720
PRJEB67810	ERR12422898
PRJEB67810	ERR12422721
PRJEB67810	ERR12422903
PRJEB67810	ERR12422726
PRJEB67810	ERR12422905

PRJEB67810	ERR12422729
PRJEB67810	ERR12422906
PRJEB67810	ERR12422908
PRJEB67810	ERR12422739
PRJEB67810	ERR12422912
PRJEB67810	ERR12422740
PRJEB67810	ERR12422914
PRJEB67810	ERR12422742
PRJEB67810	ERR12422917
PRJEB67810	ERR12422924
PRJEB67810	ERR12422746
PRJEB67810	ERR12422930
PRJEB67810	ERR12422934
PRJEB67810	ERR12422756
PRJEB67810	ERR12422758
PRJEB67810	ERR12422936
PRJEB67810	ERR12422938
PRJEB67810	ERR12422759
PRJEB67810	ERR12422939
PRJEB67810	ERR12422771
PRJEB67810	ERR12422940
PRJEB67810	ERR12422943
PRJEB67810	ERR12422772
PRJEB67810	ERR12422774
PRJEB67810	ERR12422780
PRJEB67810	ERR12422944
PRJEB67810	ERR12422947
PRJEB67810	ERR12422781
PRJEB67810	ERR12422948
PRJEB67810	ERR12422790
PRJEB67810	ERR12422792
PRJEB67810	ERR12422953
PRJEB67810	ERR12422954
PRJEB67810	ERR12422793
PRJEB67810	ERR12422955
PRJEB67810	ERR12422794
PRJEB67810	ERR12422957
PRJEB67810	ERR12422797
PRJEB67810	ERR12422958
PRJEB67810	ERR12422798
PRJEB67810	ERR12422960
PRJEB67810	ERR12422800
PRJEB67810	ERR12422965
PRJEB67810	ERR12422801
PRJEB67810	ERR12422968
PRJEB67810	ERR12422805
PRJEB67810	ERR12422969
PRJEB67810	ERR12422808
PRJEB67810	ERR12422809
PRJEB67810	ERR12422972
PRJEB67810	ERR12422812

PRJEB67810	ERR12422975
PRJEB67810	ERR12422815
PRJEB67810	ERR12422977
PRJEB67810	ERR12422980
PRJEB67810	ERR12422817
PRJEB67810	ERR12422981
PRJEB67810	ERR12422822
PRJEB67810	ERR12422825
PRJEB67810	ERR12422984
PRJEB67810	ERR12422989
PRJEB67810	ERR12422992
PRJEB67810	ERR12422827
PRJEB67810	ERR12422837
PRJEB67810	ERR12422996
PRJEB67810	ERR12422840
PRJEB67810	ERR12422998
PRJEB67810	ERR12422999
PRJEB67810	ERR12422843
PRJEB67810	ERR12423002
PRJEB67810	ERR12422850
PRJEB67810	ERR12423003
PRJEB67810	ERR12423004
PRJEB67810	ERR12423006
PRJEB67810	ERR12423009
PRJEB67810	ERR12422856
PRJEB67810	ERR12423011
PRJEB67810	ERR12422859
PRJEB67810	ERR12422865
PRJEB67810	ERR12422866
PRJEB67810	ERR12423014
PRJEB67810	ERR12423016
PRJEB67810	ERR12422869
PRJEB67810	ERR12423018
PRJEB67810	ERR12422870
PRJEB67810	ERR12422873
PRJEB67810	ERR12422875
PRJEB67810	ERR12423024
PRJEB67810	ERR12422877
PRJEB67810	ERR12422881
PRJEB67810	ERR12423028
PRJEB67810	ERR12423031
PRJEB67810	ERR12423033
PRJEB67810	ERR12423035
PRJEB67810	ERR12422884
PRJEB67810	ERR12422887
PRJEB67810	ERR12423036
PRJEB67810	ERR12422889
PRJEB67810	ERR12422891
PRJEB67810	ERR12422893
PRJEB67810	ERR12423039
PRJEB67810	ERR12423041

PRJEB67810	ERR12422894
PRJEB67810	ERR12423042
PRJEB67810	ERR12422897
PRJEB67810	ERR12422899
PRJEB67810	ERR12423047
PRJEB67810	ERR12422904
PRJEB67810	ERR12423048
PRJEB67810	ERR12422907
PRJEB67810	ERR12423054
PRJEB67810	ERR12423055
PRJEB67810	ERR12422910
PRJEB67810	ERR12423058
PRJEB67810	ERR12423059
PRJEB67810	ERR12423063
PRJEB67810	ERR12422911
PRJEB67810	ERR12423066
PRJEB67810	ERR12422913
PRJEB67810	ERR12423068
PRJEB67810	ERR12422915
PRJEB67810	ERR12423072
PRJEB67810	ERR12422922
PRJEB67810	ERR12423079
PRJEB67810	ERR12423080
PRJEB67810	ERR12423081
PRJEB67810	ERR12423084
PRJEB67810	ERR12423085
PRJEB67810	ERR12422928
PRJEB67810	ERR12423087
PRJEB67810	ERR12422931
PRJEB67810	ERR12423092
PRJEB67810	ERR12422933
PRJEB67810	ERR12423094
PRJEB67810	ERR12422935
PRJEB67810	ERR12422937
PRJEB67810	ERR12423095
PRJEB67810	ERR12423096
PRJEB67810	ERR12422941
PRJEB67810	ERR12422950
PRJEB67810	ERR12423099
PRJEB67810	ERR12423104
PRJEB67810	ERR12422952
PRJEB67810	ERR12422961
PRJEB67810	ERR12423107
PRJEB67810	ERR12423108
PRJEB67810	ERR12422962
PRJEB67810	ERR12422970
PRJEB67810	ERR12423114
PRJEB67810	ERR12422974
PRJEB67810	ERR12423115
PRJEB67810	ERR12422976
PRJEB67810	ERR12423116

PRJEB67810	ERR12422982
PRJEB67810	ERR12423117
PRJEB67810	ERR12422983
PRJEB67810	ERR12422986
PRJEB67810	ERR12423119
PRJEB67810	ERR12422993
PRJEB67810	ERR12422995
PRJEB67810	ERR12423121
PRJEB67810	ERR12422997
PRJEB67810	ERR12423123
PRJEB67810	ERR12423127
PRJEB67810	ERR12423001
PRJEB67810	ERR12423128
PRJEB67810	ERR12423013
PRJEB67810	ERR12423017
PRJEB67810	ERR12423132
PRJEB67810	ERR12423021
PRJEB67810	ERR12423022
PRJEB67810	ERR12423136
PRJEB67810	ERR12423023
PRJEB67810	ERR12423025
PRJEB67810	ERR12423140
PRJEB67810	ERR12423151
PRJEB67810	ERR12423026
PRJEB67810	ERR12423153
PRJEB67810	ERR12423029
PRJEB67810	ERR12423030
PRJEB67810	ERR12423155
PRJEB67810	ERR12423032
PRJEB67810	ERR12423034
PRJEB67810	ERR12423159
PRJEB67810	ERR12423166
PRJEB67810	ERR12423040
PRJEB67810	ERR12423043
PRJEB67810	ERR12423167
PRJEB67810	ERR12423168
PRJEB67810	ERR12423044
PRJEB67810	ERR12423177
PRJEB67810	ERR12423181
PRJEB67810	ERR12423045
PRJEB67810	ERR12423183
PRJEB67810	ERR12423049
PRJEB67810	ERR12423052
PRJEB67810	ERR12423187
PRJEB67810	ERR12423053
PRJEB67810	ERR12423188
PRJEB67810	ERR12423189
PRJEB67810	ERR12423190
PRJEB67810	ERR12423191
PRJEB67810	ERR12423194
PRJEB67810	ERR12423195

PRJEB67810	ERR12423197
PRJEB67810	ERR12423208
PRJEB67810	ERR12423209
PRJEB67810	ERR12423218
PRJEB67810	ERR12423057
PRJEB67810	ERR12423220
PRJEB67810	ERR12423221
PRJEB67810	ERR12423062
PRJEB67810	ERR12423224
PRJEB67810	ERR12423065
PRJEB67810	ERR12423069
PRJEB67810	ERR12423070
PRJEB67810	ERR12423227
PRJEB67810	ERR12423230
PRJEB67810	ERR12423075
PRJEB67810	ERR12423077
PRJEB67810	ERR12423232
PRJEB67810	ERR12423234
PRJEB67810	ERR12423082
PRJEB67810	ERR12423237
PRJEB67810	ERR12423239
PRJEB67810	ERR12423090
PRJEB67810	ERR12423093
PRJEB67810	ERR12423240
PRJEB67810	ERR12423101
PRJEB67810	ERR12423103
PRJEB67810	ERR12423245
PRJEB67810	ERR12423247
PRJEB67810	ERR12423105
PRJEB67810	ERR12423106
PRJEB67810	ERR12423249
PRJEB67810	ERR12423259
PRJEB67810	ERR12423111
PRJEB67810	ERR12423260
PRJEB67810	ERR12423112
PRJEB67810	ERR12423264
PRJEB67810	ERR12423122
PRJEB67810	ERR12423267
PRJEB67810	ERR12423124
PRJEB67810	ERR12423125
PRJEB67810	ERR12423269
PRJEB67810	ERR12423126
PRJEB67810	ERR12423271
PRJEB67810	ERR12423274
PRJEB67810	ERR12423131
PRJEB67810	ERR12423275
PRJEB67810	ERR12423278
PRJEB67810	ERR12423133
PRJEB67810	ERR12423279
PRJEB67810	ERR12423135
PRJEB67810	ERR12423137

PRJEB67810	ERR12423280
PRJEB67810	ERR12423142
PRJEB67810	ERR12423281
PRJEB67810	ERR12423147
PRJEB67810	ERR12423149
PRJEB67810	ERR12423283
PRJEB67810	ERR12423287
PRJEB67810	ERR12423150
PRJEB67810	ERR12423289
PRJEB67810	ERR12423291
PRJEB67810	ERR12423156
PRJEB67810	ERR12423292
PRJEB67810	ERR12423157
PRJEB67810	ERR12423294
PRJEB67810	ERR12423160
PRJEB67810	ERR12423296
PRJEB67810	ERR12423299
PRJEB67810	ERR12423163
PRJEB67810	ERR12423301
PRJEB67810	ERR12423303
PRJEB67810	ERR12423164
PRJEB67810	ERR12423307
PRJEB67810	ERR12423169
PRJEB67810	ERR12423314
PRJEB67810	ERR12423174
PRJEB67810	ERR12423321
PRJEB67810	ERR12423324
PRJEB67810	ERR12423175
PRJEB67810	ERR12423176
PRJEB67810	ERR12423325
PRJEB67810	ERR12423180
PRJEB67810	ERR12423331
PRJEB67810	ERR12423182
PRJEB67810	ERR12423184
PRJEB67810	ERR12423333
PRJEB67810	ERR12423196
PRJEB67810	ERR12423335
PRJEB67810	ERR12423198
PRJEB67810	ERR12423199
PRJEB67810	ERR12423340
PRJEB67810	ERR12423200
PRJEB67810	ERR12423349
PRJEB67810	ERR12423201
PRJEB67810	ERR12423202
PRJEB67810	ERR12423204
PRJEB67810	ERR12423206
PRJEB67810	ERR12423211
PRJEB67810	ERR12423212
PRJEB67810	ERR12423213
PRJEB67810	ERR12423215
PRJEB67810	ERR12423219

PRJEB67810	ERR12423222
PRJEB67810	ERR12423226
PRJEB67810	ERR12423233
PRJEB67810	ERR12423238
PRJEB67810	ERR12423241
PRJEB67810	ERR12423246
PRJEB67810	ERR12423250
PRJEB67810	ERR12423251
PRJEB67810	ERR12423252
PRJEB67810	ERR12423254
PRJEB67810	ERR12423257
PRJEB67810	ERR12423266
PRJEB67810	ERR12423270
PRJEB67810	ERR12423284
PRJEB67810	ERR12423285
PRJEB67810	ERR12423290
PRJEB67810	ERR12423293
PRJEB67810	ERR12423295
PRJEB67810	ERR12423298
PRJEB67810	ERR12423300
PRJEB67810	ERR12423304
PRJEB67810	ERR12423306
PRJEB67810	ERR12423309
PRJEB67810	ERR12423310
PRJEB67810	ERR12423311
PRJEB67810	ERR12423313
PRJEB67810	ERR12423315
PRJEB67810	ERR12423316
PRJEB67810	ERR12423318
PRJEB67810	ERR12423319
PRJEB67810	ERR12423320
PRJEB67810	ERR12423322
PRJEB67810	ERR12423323
PRJEB67810	ERR12423326
PRJEB67810	ERR12423327
PRJEB67810	ERR12423329
PRJEB67810	ERR12423336
PRJEB67810	ERR12423338
PRJEB67810	ERR12423341
PRJEB67810	ERR12423342
PRJEB67810	ERR12423345
PRJEB67810	ERR12423346
PRJEB67810	ERR12423347
PRJEB89252	ERR16073701
PRJEB89252	ERR16073702
PRJEB89252	ERR16073703
PRJEB89252	ERR16073704
PRJEB89252	ERR16073705
PRJEB89252	ERR16073706
PRJEB89252	ERR16073707
PRJEB89252	ERR16073708

PRJEB89252	ERR16073709
PRJEB89252	ERR16073710
PRJEB89252	ERR16073711
PRJEB89252	ERR16073712
PRJEB89252	ERR16073713
PRJEB89252	ERR16073714
PRJEB89252	ERR16073715
PRJEB89252	ERR16073716
PRJEB89252	ERR16073717
PRJEB89252	ERR16073718
PRJEB89252	ERR16073719
PRJEB89252	ERR16073720
PRJEB89252	ERR16073721
PRJEB89252	ERR16073722
PRJEB89252	ERR16073723
PRJEB89252	ERR16073724
PRJEB89252	ERR16073725
PRJEB89252	ERR16073726
PRJEB89252	ERR16073727
PRJEB89252	ERR16073728
PRJEB89252	ERR16073729
PRJEB89252	ERR16073730
PRJEB89252	ERR16073731
PRJEB89252	ERR16073732
PRJEB89252	ERR16073733
PRJEB89252	ERR16073734
PRJEB89252	ERR16073735
PRJEB89252	ERR16073736
PRJEB89252	ERR16073737
PRJEB89252	ERR16073738
PRJEB89252	ERR16073739
PRJEB89252	ERR16073740
PRJEB89252	ERR16073741
PRJEB89252	ERR16073742
PRJEB89252	ERR16073743
PRJEB89252	ERR16073744
PRJEB89252	ERR16073745
PRJEB89252	ERR16073746
PRJEB89252	ERR16073747
PRJEB89252	ERR16073748
PRJEB89252	ERR16073749
PRJEB89252	ERR16073750
PRJEB89252	ERR16073751
PRJEB89252	ERR16073752
PRJEB89252	ERR16073753
PRJEB89252	ERR16073754
PRJEB89252	ERR16073755
PRJEB89252	ERR16073756
PRJEB89252	ERR16073757
PRJEB89252	ERR16073758
PRJEB89252	ERR16073759

PRJEB89252	ERR16073760
PRJEB89252	ERR16073761
PRJEB89252	ERR16073762
PRJEB89252	ERR16073763
PRJEB89252	ERR16073764
PRJEB89252	ERR16073765
PRJEB89252	ERR16073766
PRJEB89252	ERR16073767
PRJEB89252	ERR16073768
PRJEB89252	ERR16073769
PRJEB89252	ERR16073770
PRJEB89252	ERR16073771
PRJEB89252	ERR16073772
PRJEB89252	ERR16073773
PRJEB89252	ERR16073774
PRJEB89252	ERR16073775
PRJEB89252	ERR16073776
PRJEB89252	ERR16073777
PRJEB89252	ERR16073778
PRJEB89252	ERR16073779
PRJEB89252	ERR16073780
PRJEB89252	ERR16073781
PRJEB89252	ERR16073782
PRJEB89252	ERR16073783
PRJEB89252	ERR16073784
PRJEB89252	ERR16073785
PRJEB89252	ERR16073786
PRJEB89252	ERR16073787
PRJEB89252	ERR16073788
PRJEB89252	ERR16073789
PRJEB89252	ERR16073790
PRJEB89252	ERR16073791
PRJEB89252	ERR16073792
PRJEB89252	ERR16073793
PRJEB89252	ERR16073794
PRJEB89252	ERR16073795
PRJEB89252	ERR16073796
PRJEB89252	ERR16073797
PRJEB89252	ERR16073798
PRJEB89252	ERR16073799
PRJEB89252	ERR16073800
PRJEB89252	ERR16073801
PRJEB89252	ERR16073802
PRJEB89252	ERR16073803
PRJEB89252	ERR16073804
PRJEB89252	ERR16073805
PRJEB89252	ERR16073806
PRJEB89252	ERR16073807
PRJEB89252	ERR16073808
PRJEB89252	ERR16073809
PRJEB89252	ERR16073810

PRJEB89252	ERR16073811
PRJEB89252	ERR16073812
PRJEB89252	ERR16073813
PRJEB89252	ERR16073814

Figure 2 script

```
library(ComplexHeatmap)
library(circlize)
library(dendextend)
library(dplyr)
library(tibble)
library(grid)
library(RColorBrewer)

# --- clustering ---
dist_mat <- as.dist(1 - mat)
hc <- hclust(dist_mat, method = "average")

# --- choose number of clusters ---
k_clusters <- 13

# --- clusters ---
clusters <- cutree(hc, k = k_clusters)
names(clusters) <- rownames(mat)

# --- dynamic colours ---
my_colors <- c(
  "#E41A1C", "#377EB8", "#8D6E63", "#984EA3",
  "#31D492", "#2D9966", "#F781BF", "#546E7A",
  "#FF8904", "#A684FF","#42D3F2", "#90A4AE","#0C0A09"
)

cluster_colors <- setNames(my_colors[1:k_clusters], sort(unique(clusters)))
label_colors <- cluster_colors[as.character(clusters[rownames(mat)])]
```

```
stopifnot(length(label_colors) == nrow(mat))

# --- metadata alignment ---
meta <- meta[rownames(mat), , drop = FALSE]

# --- annotations with cluster colours ---
row_anno <- rowAnnotation(
  spacer = anno_empty(width = unit(0.1, "mm"), border = FALSE),

  "Host ST" = anno_text(
    meta$`Host MLST`,
    gp = gpar(fontsize = 12, col = label_colors, fontface = "bold"),
    just = "left"
  ),

  "blaCTX-M" = anno_text(
    meta$`blaCTX-M`,
    gp = gpar(fontsize = 12, col = label_colors, fontface = "bold"),
    just = "left"
  ),

  "Inc-type" = anno_text(
    meta$`Inc type`,
    gp = gpar(fontsize = 12, col = label_colors, fontface = "bold"),
    just = "left"
  ),

  gap = unit(5, "mm")
)

# --- heatmap ---
ht <- Heatmap(
  mat,
  name = "Similarity",
```

```
  col = colorRamp2(seq(0, 1, length.out = 9), brewer.pal(9, "YlGnBu")),
  cluster_rows = hc,
  cluster_columns = hc,
  show_row_names = TRUE,
  row_names_side = "left",
  row_names_gp = gpar(fontsize = 12, col = label_colors, fontface = "bold"),
  row_names_max_width = unit(5, "cm"),
  row_dend_width = unit(6, "cm"),
  show_column_dend = FALSE,
  show_column_names = FALSE,
  left_annotation = row_anno,

  width = unit(25, "cm"),
  show_heatmap_legend = FALSE

)

lgd <- Legend(
  title = "Similarity",
  col_fun = colorRamp2(seq(0, 1, length.out = 9), brewer.pal(9, "YlGnBu")),
  legend_height = unit(10, "cm"),
  grid_width = unit(0.5, "cm"),
  labels_gp = gpar(fontsize = 12,fontface = "bold"),
  # --- Title Centering Settings ---
  title_gp = gpar(fontsize = 14, fontface = "bold"),
  title_position = "topcenter",
  title_gap = unit(4, "mm")
)

lgd_list <- packLegend(lgd)

jpeg("heatmap.jpeg", width = 20, height = 12, units = "in", res = 800)
draw(ht, padding = unit(c(2, 2, 11, 2), "mm"))
draw(lgd, x = unit(0.935, "npc"), y = unit(0.5, "npc"), just = "left")
```

```
# --- headers ---
grid.text("Host ST", x = unit(0.318, "npc"), y = unit(0.97, "npc"),
        gp = gpar(fontface = "bold", fontsize = 12))

grid.text("blaCTX-M", x = unit(0.357, "npc"), y = unit(0.97, "npc"),
        gp = gpar(fontface = "bold", fontsize = 12))

grid.text("Inc-type", x = unit(0.4, "npc"), y = unit(0.97, "npc"),
        gp = gpar(fontface = "bold", fontsize = 12))
dev.off()
```
